# Multi-Scales Analysis of Primate Diversity and Protected Areas at a Megadiverse Region

**DOI:** 10.1371/journal.pone.0105205

**Published:** 2014-08-18

**Authors:** Míriam Plaza Pinto, José de Sousa e. Silva-Júnior, Adriana Almeida de Lima, Carlos Eduardo Viveiros Grelle

**Affiliations:** 1 Programa de Pós-Graduação em Ecologia, Universidade Federal do Rio de Janeiro, Rio de Janeiro, Rio de Janeiro, Brazil; 2 Coordenação de Zoologia, Museu Paraense Emilio Goeldi, Belém, Pará, Brazil; 3 Departamento de Ecologia, Universidade Federal do Rio Grande do Norte, Natal, Rio Grande do Norte, Brazil; 4 Departamento de Ecologia, Universidade Federal do Rio de Janeiro, Rio de Janeiro, Rio de Janeiro, Brazil; 5 Laboratório de Vertebrados, Universidade Federal do Rio de Janeiro, Rio de Janeiro, Rio de Janeiro, Brazil; Institut Pluridisciplinaire Hubert Curien, France

## Abstract

In this paper, we address the question of what proportion of biodiversity is represented within protected areas. We assessed the effectiveness of different protected area types at multiple scales in representing primate biodiversity in the Brazilian Legal Amazon. We used point locality data and distribution data for primate species within 1°, 0.5°, and 0.25° spatial resolution grids, and computed the area of reserves within each cell. Four different approaches were used – no reserves (A), exclusively strict use reserves (B), strict and sustainable use reserves (C), and strict and sustainable use reserves and indigenous lands (D). We used the complementarity concept to select reserve networks. The proportions of cells that were classified as reserves at a grid resolution of 1° were 37%, 64%, and 88% for approaches B, C and D, respectively. Our comparison of these approaches clearly showed the effect of an increase in area on species representation. Representation was consistently higher at coarser resolutions, indicating the effect of grain size. The high number of irreplaceable cells for selected networks identified based on approach A could be attributed to the use of point locality occurrence data. Although the limited number of point occurrences for some species may have been due to a Wallacean shortfall, in some cases it may also be the result of an actual restricted geographic distribution. The existing reserve system cannot be ignored, as it has an established structure, legal protection status, and societal recognition, and undoubtedly represents important elements of biodiversity. However, we found that strict use reserves (which are exclusively dedicated to biodiversity conservation) did not effectively represent primate species. This finding may be related to historical criteria for selecting reserves based on political, economic, or social motives.

## Introduction

The Amazon is the largest Brazilian biome and has been subjected to several destructive changes during the last decade [1]. It contains over half of all global tropical forest and provides habitat for a wide range of biodiversity [2]. Considering the magnitude of loss of habitat and general ecological relationships (for example species area relationships), some studies predict that an extinction debt will have to be paid in the future [3], [4].

Deforestation itself may not be the only cause of threats to species (see [5] for an example relating to Amazonian mammals). It has consequences other than habitat area reduction, such as fragmentation [6] and selective logging [7]. Selective logging is spatially diffuse and difficult to monitor [8]. Other indirect effects include hunting [9] and fire [7], [10], [11], since deforestation benefits hunters by increasing an area's accessibility [12], while border effects, heat, and desiccation are conducive to the occurrence of fire [13], [14]. Other factors such as invasive exotic species [15] are a further threat to the persistence of native species. Some threats, such as agriculture, hunting, and rural or urban expansion, act synergistically [16] in affecting tropical mammal species [14]. When there are several competing demands on land use, it is important to prioritize areas with the specific purpose of conserving biological diversity.

Protected areas cover 12.9% of the global terrestrial area, of which 5.8% is in reserves with strict use regulation (IUCN categories I–IV) [17]. Latin America contains more sustainable use reserves (20%; categories V and VI) as well as non-categorized reserves than any other continent [18]. A total of 11.1% of the entire area of the Amazon is strictly protected [19]. Protected areas are heterogeneously distributed among the Brazilian biomes, of which the Amazon has the greatest proportion of protected areas. Regarding categories of protection, 5.7% of its area is under strict use regulation, 1.9% is allocated to sustainable use reserves, and 17.7% to indigenous land [20].

Protected areas are spatially correlated with low deforestation and selective logging rates in humid tropical forests [2]. For example, deforestation and selective logging in protected areas are lower compared to adjacent lands in a region with rapid agro-industrialization rates south of the Brazilian Amazon [7]. Natural and indigenous reserves constitute refuges for tropical biodiversity, featuring fewer incidences of deforestation and fire [21]. These areas are also generally protected against selective logging [8].

It is therefore assumed that protected areas secure biodiversity against several threats. However, there remains the question of the proportion of biodiversity that is represented within protected areas. An assessment of the extent to which conservation targets have already been achieved in existing conservation areas is of critical importance in this regard [22]. Gap analysis is a process for spatially comparing data on species or other biodiversity elements (especially conservation-related data) under various types of land use, to identify gaps in protection [23]–[25]. Several systematic conservation-planning studies have assessed how effective existing reserves are at representing specific biodiversity elements [26]–[29]. These include recent studies on South American mammals [30] and endemic Atlantic Forest primates [31].

Threats to the Amazon affect primate species of this biome in different ways [32]. The Amazon is the region with the greatest primate diversity in South America. There are 92 primate species occurring in the Brazilian Amazon [33], of which 26 are on the Brazilian list of threatened species [34]. Several species have narrow ranges [35]. Rivers play an important role in restricting dispersion of Amazonian primates, while interspecific differences in the size of geographic distribution areas may be associated with the capacity of different species to transcend these barriers [36]. Primates are a well-studied group relative to other mammals, likely because most of them are visible, diurnal, have a large body size, and are arboreal, noisy and colorful [37].

Our aim was to assess the effectiveness of current protected areas in representing primate diversity in the Brazilian Amazon using grids of different resolutions. This evaluation considered strict and sustainable use reserves and indigenous lands, as well as different combinations thereof. Strict use reserves are areas dedicated exclusively to nature conservation, whereas sustainable reserves aim to reconcile nature conservation and sustainable use of natural resources. Indigenous lands are socio-cultural areas that are not explicitly created with the purpose of conserving biological diversity, but nevertheless seem to contribute to this end [8], [21]. For this study, we constructed a database of point occurrence data and we also used the IUCN distribution database [38]. A minimum area criterion was used to classify a grid cell as a reserve. We also mapped irreplaceability patterns indicating priority regions needed to guarantee representation of all species.

## Materials and Methods

**Figure 1:**
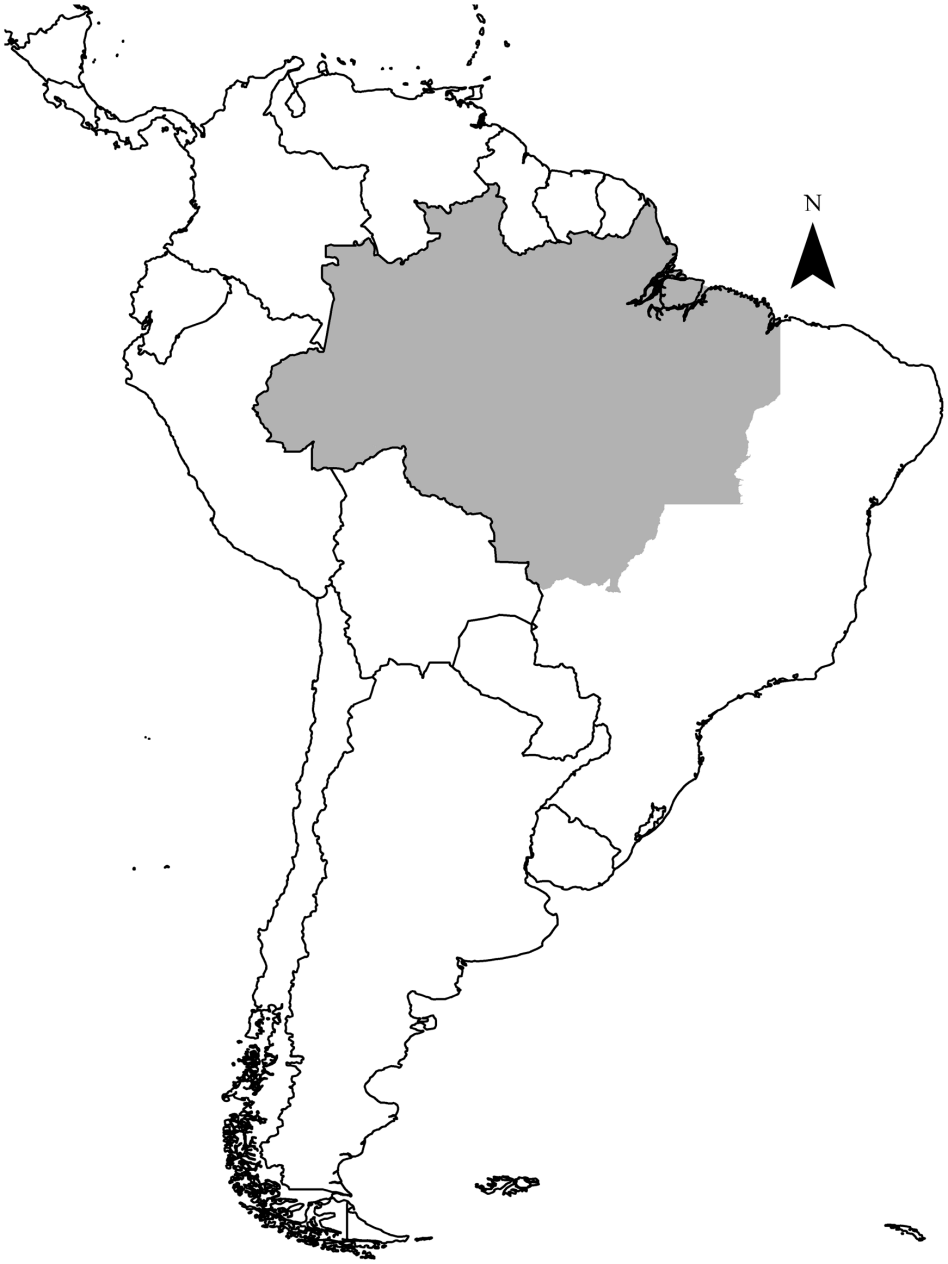
Legal Amazon region, South America. The Legal Amazon covers a greater part of the biome, and is located inside Brazilian borders. It is a politically categorized area used by the Brazilian government for planning, coordinating, controlling and executing actions for regional development.

We constructed a database with locality data for all primate species occurring within Legal Amazon in Brazil (Figure 1). Legal Amazon is a politically categorized area used by the Brazilian government for planning, coordinating, controlling, and executing actions for regional development, which encompasses this biome (see http://www.sudam.gov.br/amazonia-legal). Locality data were initially compiled from the Museu Paraense Emílio Goeldi (MPEG) mammal collection. Using the Web of Science (http://apps.isiknowledge.com) and Scielo (http://www.scielo.br/) databases, we searched for articles published between 2000 and 2013 (last update) using each genus name as a separate keyword. We also searched the *Neotropical Primates* and *Checklist* journals that were not indexed in these databases. Locality data for primate species recorded by experts (primate specialists) were extracted from these articles. Consulted articles are listed in Text S1, including specific reviews for each genus. Some localities were georeferenced using Global Gazetteer Version 2.1 (http://www.fallingrain.com/world, last accessed in December 2009), National Geospatial-Intelligence Agency (http://www.nga.mil/portal/site/nga01/, last accessed in December 2009), and the speciesLink project (http://splink.cria.org.br/, last accessed in December 2009). Specific details for each genus can be found in Text S2. Primate species distribution data, outlining estimated ranges for each species, were obtained from IUCN [38].

We conducted our analysis at multiple scales using three grids. The first was composed of 433 cells at a spatial resolution of 1° latitude/longitude, the second of 1,687 cells at a resolution of 0.5°, and the third of 6,667 cells at a resolution of 0.25°. These grids were superimposed on the Legal Amazon map (IBGE <http://downloads.ibge.gov.br/downloads_geociencias.htm>, accessed in October 2009). To be included in the analysis, cells had to overlap with Legal Amazon by at least 25%. This ensured that at least 25% of each grid cell was part of the Amazon biome. All databases were incorporated in this grid, and the cells were considered the units for data analysis.

Maps of strict use reserves (IUCN categories I–IV) and sustainable use reserves (IUCN categories V and VI) were obtained from the Ministry of Environment (Ministério do Meio Ambiente - MMA) database (http://mapas.mma.gov.br/i3geo/datadownload.htm, accessed in January 2014). Indigenous lands maps were obtained from the National Indian Foundation (Fundação Nacional do Índio - FUNAI) database (http://mapas.funai.gov.br/). We computed the area of each protected area type (strict use, sustainable use, and indigenous land) within each cell. Cells that included 11,570 ha or more of protected areas were categorized as reserves (see below).

Our decision about the minimum area used to designate a cell as a reserve was based on a population viability analysis (PVA) previously conducted for *Brachyteles* [39], an Atlantic Forest genus. However, no PVA analysis has to date been carried out for a large Amazonian primate species. We assumed that the area needed to maintain viable populations was positively correlated to body size [40]. The species belonging to the *Brachyteles* genus have individual body weights of 9.4–12.1 kg [40]. The largest Amazonian primate genera include *Alouatta*, *Ateles*, and *Lagothrix* with weight ranges of 3.8–9.0 kg, 7.0–9.0 kg, and 7.0–12.0 kg, respectively [41].

Selection of priority areas was performed using the following four approaches:

- For this approach, the existence of actual reserves in the Legal Amazon was ignored.
- All cells with at least 11,570 ha of existing strict use protected areas were classified as reserves. These cells were automatically included in the network, and the remaining priority areas were selected taking these pre-existing reserves into account.
- As B), including sustainable use protected areas.
- As C), including indigenous lands.

We used gap analysis to ascertain whether the cells in which each species occurred coincided with existing reserves (for approaches B, C, and D). If there were no matches, the species was considered not to be represented in the existing reserve system. Gap analysis was also done using distribution data.

The reserve selection procedure was carried out using MARXAN software to implement a simulated annealing algorithm [42]–[45]. For each of the four approaches we performed 200 runs with 10^6^ iterations. Unless each species occurs in only one cell, multiple solutions exist for achieving the goal of representing all species within the minimum number of cells. A high number of interactions increases the likelihood of finding good solutions (a minimum number of cells representing each of the species at least once) using the simulated annealing algorithm. We used an irreplaceability index for the top 100 best solutions. This index was calculated by summing the number of times each cell appeared in a solution, using a minimum value of 0 for cells that did not appear in any solution and a maximum value of 100 for cells that appeared in all solutions.

To test the effectiveness of the existing network of reserves relative to species representation, we randomly selected 10^4^ networks composed of the same number of cells considered as reserves (for each approach B, C, and D). The frequency distribution of the number of primate species represented in the random networks was compared with the actual number of species represented in the reserve cells. Random networks were selected using a script written in R [46].

## Results

**Figure 2:**
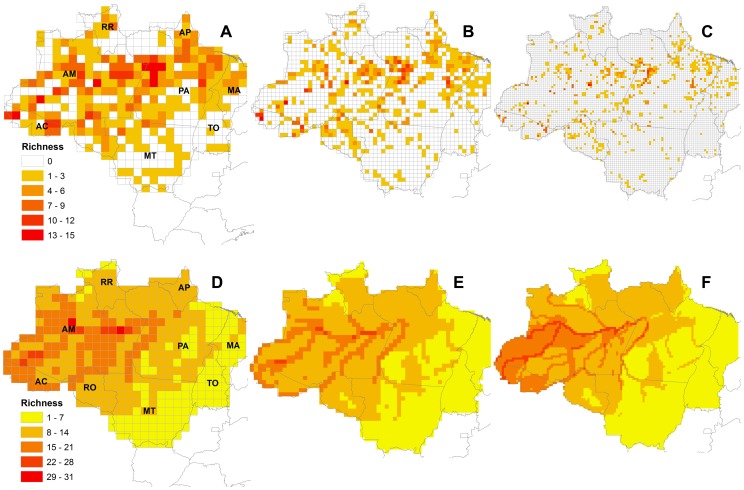
Spatial richness pattern of Amazonian primate species. Number of primate species occurring in each grid cell in the 1^o^ grid, with a total of 433 cells (A and D), the 0.5^o^ grid, with a total of 1687 cells (B and E), and the 0.25^o^ grid, with a total of 6,667 cells (C and F) in Legal Amazon, based on point locality occurrence data (A, B and C) and distribution data (D, E and F). States are indicated in A and D as follows. AC – Acre, AM – Amazonas, AP – Amapá, MA – Maranhão, MT – Mato Grosso, PA – Pará, RO –Rondônia, RR – Roraima, TO – Tocantins.

The occurrence database yielded 1,690 localities for 87 primate species grouped within 16 genera in the Legal Amazon area. The following species each occurred in a single 0.25^o^ grid cells: *Aotus vociferans* (Spix, 1823), *Callicebus regulus* Thomas, 1927, *Mico manicorensis* Van Roosmalen, Van Roosmalen, Mittermeier & Rylands, 2000, and *Mico marcai* (Alperin, 1993). The species with the highest number of occurrences in different cells was *Sapajus apella* (Linnaeus, 1758). For several species the number of point occurrences did not correspond to the number of cells of the grid in which they occurred, as some points were spatially grouped. Numbers of cells containing occurrence data for primate species at 0.25°, 0.5°, and 1° were 782 (11.73%), 510 (30.23%), and 266 (61.43%), respectively (Figure 2). Cells with high richness values were well dispersed across the grids.

Numbers of cells containing at least 11,570 ha of strict use reserves (approach B), strict and sustainable use reserves (approach C), or strict and sustainable use reserves and indigenous lands (approach D) were 161 (37.18%), 275 (63.51%), and 383 (88.45%), respectively, at a grid resolution of 1° (Tables 1 and 2). Results for other grid resolutions are also shown. Cells classified as reserves were often adjacent to one another.

**Table 1:** Selection of priority areas in Legal Amazon for conservation at three resolutions, based on point locality occurrence data for primates.

		Existing Protected Areas		Primate species		Random networks	
Resolution	Approach	N	P	N	P	N	P
1^o^	B	161	37.18%	75	86.21%	3859	0.39
1^o^	C	275	63.51%	84	96.55%	1608	0.16
1^o^	D	383	88.45%	86	98.85%	3948	0.39
0.5^o^	B	348	20.63%	66	75.86%	5560	0.56
0.5^o^	C	736	43.63%	78	89.66%	5463	0.55
0.5^o^	D	1183	70.12%	82	94.25%	8670	0.87
0.25^o^	B	840	12.60%	52	59.77%	9820	0.98
0.25^o^	C	2039	30.58%	74	85.06%	6895	0.69
0.25^o^	D	3575	53.62%	79	90.80%	9359	0.94

**Resolution** gives the size of the grid cells. **Approach** gives the approach to classifying grid cells as reserves (B takes only strict use reserves into account, C uses strict use and sustainable use reserves, and D uses strict use, sustainable use and indigenous lands). Under **Existing Protected Areas**, **N** gives the number of grid cells classified as existing reserves on the basis that they contain at least 11,570 ha of existing reserves, and **P** gives the proportion that these represent of the total grid cells in Legal Amazon. Under **Primate Species**, **N** gives the number of primate species occurring in these grid cells, and **P** gives the proportion that these represent of all Amazonian primate species. Under **Random Networks**, **N** gives the number of randomly selected networks that protected more species than the existing reserve network, and **P** gives the proportion relative to 10.000 (which is the total number of random networks selected).

**Table 2:** Selection of priority areas in Legal Amazon for conservation at three resolutions, based on distribution data for primates.

		Existing Protected Areas		Primate species		Random networks	
Resolution	Approach	N	P	N	P	N	P
1^o^	B	161	37.18%	88	97.78%	9590	0.96
1^o^	C	275	63.51%	90	100.00%	0	<0.01
1^o^	D	383	88.45%	90	100.00%	0	<0.01
0.5^o^	B	348	20.63%	85	94.44%	9332	0.93
0.5^o^	C	736	43.63%	89	98.89%	5427	0.54
0.5^o^	D	1183	70.12%	89	98.89%	9030	0.90
0.25^o^	B	840	12.60%	82	91.11%	10000	1.00
0.25^o^	C	2039	30.58%	89	98.89%	6654	0.67
0.25^o^	D	3575	53.62%	89	98.89%	9433	0.94

**Resolution** gives the size of the grid cells. **Approach** gives the approach to classifying grid cells as reserves (B takes only strict use reserves into account, C uses strict use and sustainable use reserves, and D uses strict use, sustainable use and indigenous lands). Under **Existing Protected Areas**, **N** gives the number of grid cells classified as existing reserves on the basis that they contain at least 11,570 ha of existing reserves, and **P** gives the proportion that these represent of the total grid cells in Legal Amazon. Under **Primate Species**, **N** gives the number of primate species occurring in these grid cells, and **P** gives the proportion that these represent of all Amazonian primate species. Under **Random Networks**, **N** gives the number of randomly selected networks that protected more species than the existing reserve network, and **P** gives the proportion relative to 10.000 (which is the total number of random networks selected).

**Figure 3:**
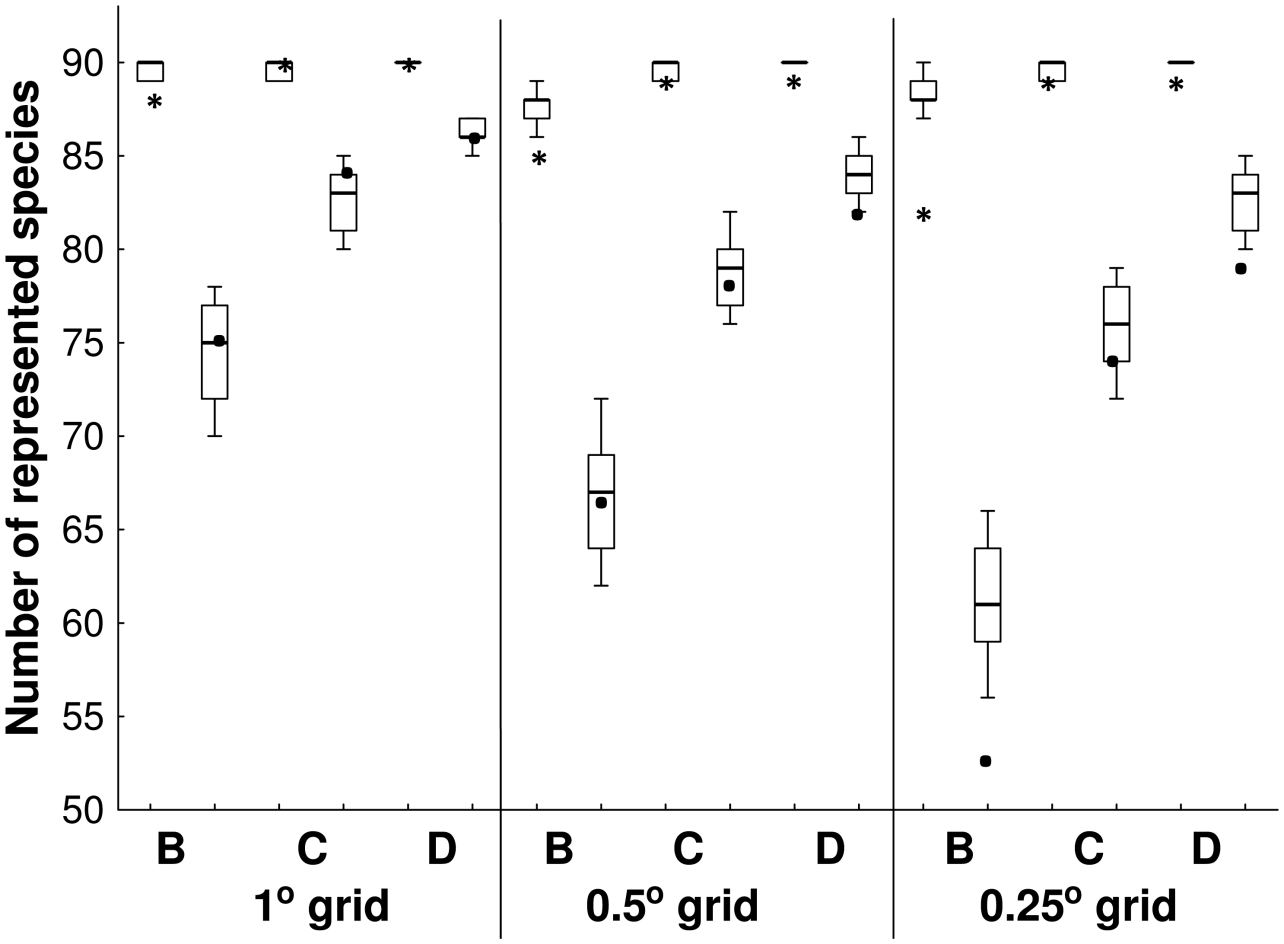
Number of primate species represented in actual and randomly selected reserve networks in Legal Amazon. Number of species represented in the existing reserve network (closed circles – point locality occurrence data; asterisks – distribution data) in relation to number represented in randomly selected networks (box and whiskers: median, quartiles and deciles). This analysis was conducted at three resolutions, using a 1^o^, 0.5^o^, and 0.25^o^ grid. In approach B, only strict use reserves were included in the existing reserve network, in C both strict and sustainable use reserves were included, and in D, strict and sustainable use reserves and indigenous lands were included.

The proportion of species represented in the current network of reserves considering occurrence data varied from 60% at a grid resolution of 0.25°, considering only strict use reserves, to 99% at a grid resolution of 1°, considering strict and sustainable use reserves and indigenous lands (Table 1). When using distribution data, the proportion of species represented varied from 91% at a grid resolution of 0.25°, considering only strict use reserves, to 100% at a grid resolution of 1° considering strict and sustainable use reserves (including the indigenous lands made no difference) (Table 2). Regardless of grid resolution, the number of species represented was higher when all types of reserves were included (Table 1, Figure 3). When using distribution data, the number of species represented was exactly the same for approaches C and D, i.e. including indigenous lands made no difference (Table 2). The number of species represented was not higher than expected by chance alone (Table 1). At a grid resolution of 1°, the number of species represented in protected areas was higher than the upper quartile of random networks when considering strict use and sustainable use reserves. When all types of protected areas or only strict use reserves were considered, the number of species represented was lower than the lower decile of species in random networks using a grid resolution of 0.25° (Figure 3).

**Figure 4:**
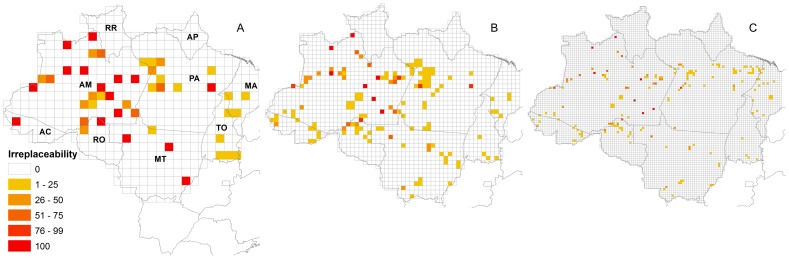
Identification of important grid cells for Amazonian primate conservation, ignoring existing reserves. Irreplaceability pattern was obtained using the 100 best network solutions for primate conservation in Legal Amazon. Grid cells were identified based on point locality occurrence data for primate species, ignoring existing reserves. Grid cells are colour coded according to the irreplaceability pattern, which is the number of solutions in which they were identified as important for primate conservation. At a grid resolution of 1^o^ (A), 24 grid cells were identified in each solution. At a resolution of 0.5^o^ (B), 31 grid cells were identified, and at 0.25^o^ (C), 32 grid cells were identified. States are indicated in A as follows: AC –Acre, AM – Amazonas, AP – Amapá, MA – Maranhão, MT – Mato Grosso, PA – Pará, RO – Rondônia, RR – Roraima, TO – Tocantins.

Networks selected in approach A that disregarded the existence of actual reserves in Legal Amazon contained 24, 31, and 32 cells at grid resolutions of 1°, 0.5°, 0.25°, respectively (Figure 4). At these resolutions, 14, 7, and 7 cells were respectively irreplaceable. Most of these irreplaceable cells were located in Amazonas, and one was at the border of the states of Amazonas and Rondônia. Some irreplaceable cells also occurred in Acre, Mato Grosso and Pará at a grid resolution of 1° (Figure 4).

**Figure 5:**
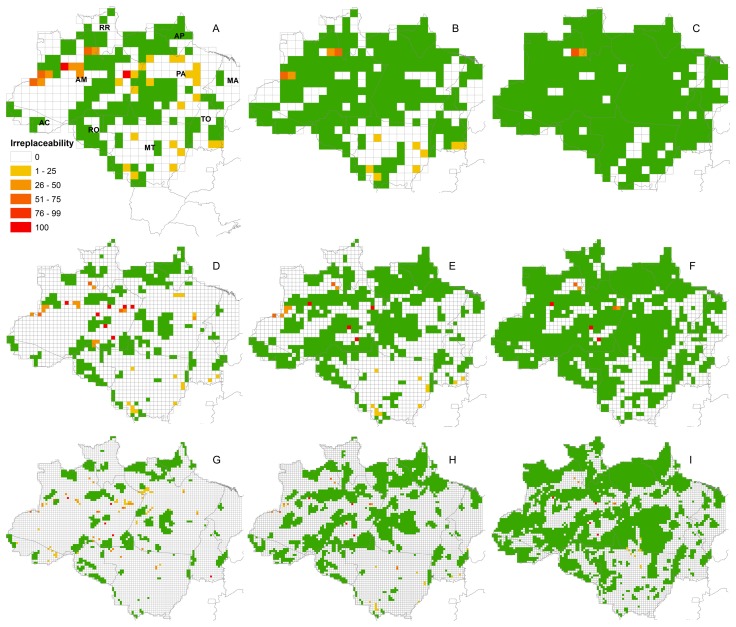
Identification of important grid cells for Amazonian primate conservation, taking existing protected areas into account. Irreplaceability pattern was obtained using the 100 best network solutions for primate conservation in Legal Amazon. Grid cells were identified based on point locality occurrence data for primate species, taking into account already existing protected areas. This process was done at three resolutions: 1^o^ (A, B and C), 0.5^o^ (D, E and F) and 0.25^o^ (G, H and I). Three categories of protected areas were used: strict use reserves (A, D and G), strict and sustainable use reserves (B, E and H), and strict and sustainable use reserves and indigenous lands (C, F and I).Grid cells are colour coded green for existing reserves, and yellow to red according to the irreplaceability pattern, which is the number of solutions in which each grid cell was identified as important, as indicated in the legend. States are indicated in A as follows: AC – Acre, AM – Amazonas, AP – Amapá, MA – Maranhão, MT – Mato Grosso, PA – Pará, RO – Rondônia, RR – Roraima, TO – Tocantins.

When previously considering only existing strict use reserves, we selected cells to complement the actual reserve system throughout the entire biome. However, the cells with higher irreplaceability values were located in Amazonas (Figure 5 A, D and G). There were no additional areas selected in states other than Amazonas when we considered other types of existing reserves at grid resolutions of 1° and 0.5° (Figure 5 B, C, E, F, H and I).

## Discussion

The existing reserve network in Legal Amazon does not completely represent the primate biodiversity of this region. Moreover, representation would probably be even lower if intraspecific diversity was considered. When we used occurrence data, representation was lower compared to random networks. The influence of an increase in area on species representation was clearly evident when we sequentially compared approaches B, C, and D. Considering all types of reserves together consistently enhanced species representation.

There was an effect of grain size. Considering existing reserves, representation of primate species was consistently higher when we used coarse resolution grids. The generalization of one point occurrence datum to an entire cell in a large grid (1°) compared with a small one (0.25°) may have inflated species representation. This effect is not so strong when using distribution data. The proportion of cells with data was positively related to grid cell size. Beyond that, a species can be considered represented in a reserve using a coarse grid resolution, but the occurrence data may not be coincident with reserves inside a large grid cell. Differences in grid resolutions affect characterization of richness patterns [47]. At coarser spatial resolutions data are less prone to false absences [47], but at gap analysis species representation may be overestimated by false presences. The combined effect of grain size and reserve types was sometimes considerable (when including all types of reserves, 54% of 0.25° cells, 70% of 0.5° cells and 88% of 1° cells were classified as reserves). Nevertheless, the number of species represented was mostly low considering the high proportion of the biome that was protected, especially at smaller grid sizes.

Small portions of some grid cells consisted of reserves. An important question is the minimum area required to classify a cell as a reserve. In another study [31], we also considered 11,570 ha as a baseline criterion of minimum area. This value was chosen based on a population viability study for larger primate species in the Atlantic Forest biome [39]. However, as previously mentioned there is no comparative study available for any large Amazon primate species.

Other studies have also used gap analysis to evaluate the representation of several species [29]. In Brazil, such studies were recently conducted for bird species from Cerrado using modeled geographic distribution [48], and for endemic primates in the Atlantic Forest [31]. Within South America, other important primate studies have focused on the identification of hotspots [49], areas of endemism [35], [50], and areas of greater rarity [37]. The present study is therefore the first study conducted on primates in the entire Legal Amazon area using gap analysis and reserve selection.

We used both species point locality occurrence data and geographic distribution data in this study, and compared the results. The use of point locality data reduces type II or commission errors that falsely indicate the occurrence of a species at a particular place. However, it also disregards places where no sampling has been done but where the species may still occur. Thus, point locality occurrence data may be biased toward areas where more studies were carried out [51]. Compared with extrapolated geographic distribution, this type of data also makes it harder to visualize, understand, and relate diversity patterns to historic, environmental, or biological characteristics. But commission errors may be inflating species representation estimates when using distribution data. Our approach in this study was to apply spatial information at a wide scale. However, this is not a panacea for conservation planning [23], and should be used in conjunction with fine-scale studies. We achieved our purpose, which was to evaluate the effectiveness of the existing Amazonian protected areas network, focusing primarily on strict use reserves, in representing primate species biodiversity. Based on the demonstrated multi-scale effect on species representation, we strongly recommend the use of a smaller grain size to reduce commission errors if the intention is to use point locality data.

Networks selected in approach A, which ignored the current reserve system, had low flexibility. Flexibility was assessed according to the number of irreplaceable cells. These cells were imperative to the network for the purpose of representing all species. The high number of irreplaceable cells could be explained by the use of point locality occurrence data. While the limited number of point occurrences of some species may be due to a lack of spatially dispersed studies and consequently of geographic distribution knowledge (Wallacean shortfall) [52], [53], it may also be caused by actual restricted geographic species distribution. Species that restricted the flexibility of networks were mainly those occurring in a small number or just one cell (*Aotus vociferans*, *Callicebus regulus*, *Mico manicorensis*, and *Mico marcai*). A lack of sampling was evident since some of these species, e.g. *Aotus trivirgatus* (Humbolt, 1811), are expected to occur within larger areas.

A point worth emphasizing is that while a smaller network could in fact be effective in representing primate species, this does not imply that we can ignore the existing reserve system [54]. Rather, additional and complementary areas to those contained within the established system should be identified. Existing reserves already enjoy a structure, legal protection status, and recognition by society [27]. More importantly, they clearly represent important elements of biodiversity other than those that our study focused on (primates). Also, we used the criterion of just one occurrence to consider a primate species as being represented. Our findings would certainly have indicated a less effective reserve network if we had considered the occurrence of each species at more than one reserve as a criterion. This is a preliminary evaluation of primate conservation in Amazonian reserves from a multispecies perspective, and it can be considered the minimum to guarantee that each species is represented in at least one reserve. However, it is evident that an assessment of individual species' population viability requires further exploration. It is also necessary to study each species individually and to include characteristics that relate to its persistence. Moreover, it is important to guarantee reserves with low anthropogenic disturbance, as these areas may be associated with higher primate density [55]. We found that strict use reserves alone did not represent primate species more effectively than random networks. These reserves are the only type of protected area that is exclusively dedicated to biodiversity conservation.

The existing Amazonian reserve system does not effectively represent primate species. We found this evaluation may be influenced by grid resolution, with an overestimation of species representation at coarser resolutions, and by type of data, with an overestimation of species representation using distribution data. The historical selection of national reserves within the Brazilian Amazon was based on criteria other than primate conservation. For example, some focused on one or a limited number of species or vegetation types, while others were selected in an *ad hoc* manner [47], based on political, economic, or social motives, without using explicit criteria [56]. In Brazil, protected areas are biased toward low altitude areas, elevated terrains, and areas that are far from roads, urban aggregations, and agriculture-dominated areas [57]. We should continue to improve distribution knowledge and database quality for a variety of taxonomic groups [24]. A limited focus on a specific taxonomic group results in biased conclusions relating to that group, although there do exist coincidences in richness patterns of, e.g., orders of Amazonian mammals [58].

Beyond inventories, other studies are needed to improve knowledge about factors that influence population viability, differences in habitat quality, interrelations among species, environmental disturbances, and other important issues in ecology and conservation biology [23]. Field studies and planning may use concepts and tools from conservation biology applicable to fine scales to design local conservation strategies [23].

## Supporting Information

Text S1 **Articles consulted for primate locality data or taxonomic classification option.**
(DOCX)

Text S2 **Specific taxonomic details followed for each genus.**
(DOCX)
