# Supplementary material for: Multi-Scales Analysis of Primate Diversity and Protected Areas at a Megadiverse Region
Source: PLoS One. 2014 Aug 18;9(8):e105205. doi: 10.1371/journal.pone.0105205 (PMC4136851; doi:10.1371/journal.pone.0105205)
Supplement: Text S1 — Articles consulted for primate locality data or taxonomic classification option. (DOCX) [file pone.0105205.s001.docx]

**Appendix S1:**

Articles consulted for primate locality data or taxonomic classification option:

***Alouatta***

Avelar, A.A. (2007) *Ontogenia e dimorfismo sexual nas espécies de guaribas vermelhos Alouatta Lacépède, 1799 (Primates, Atelidae)*. Dissertação de mestrado, Programa de Pós-Graduação em Zoologia, Museu Paraense Emílio Goeldi, UFPA.

Bastos, H.B., Gonçalves, E.C., Ferrari, S.F., Silva, A. & Schneider, M.P.C. (2010) Genetic structure of red-handed howler monkey populations in the fragmented landscape of Eastern Brazilian Amazonia. *Genetics and Molecular Biology*, **33 (4)**, 774-780.

Bezerra, B.M., Barnett, A.A., Souto, A. & Jones, G. (2011) Ethogram and natural history of Golden-backed Uakaris (*Cacajao melanocephalus*). *International Journal of Primatology*, **32**, 46-68.

Bicca-Marques, J.C. (2003) How do howler monkeys cope with habitat fragmentation? *Primates in Fragments: Ecology and Conservation.* (ed. by L.K. Marsh), pp. 283-303. Kluwer Academic/Plenum Publishers, New York.

Bobadilla, U.L. & Ferrari, S.F. (2000) Habitat use by *Chiropotes satanas utahicki* and syntopic Platyrrhines in eastern Amazonia. *American Journal of Primatology*, **50**, 215-224.

Botelho, A.L.M., Calouro, A.M., Borges, L.H.M. & Chaves, W.A. (2012) Large and medium-sized mammals of the Humaitá Forest Reserve, southwestern Amazonia, state of Acre, Brazil. *Checklist*, **8(6)**, 1190-1195.

Boyle, S.A., Lourenço, W.C., Silva, L.R. & Smith, A.T. (2009) Travel and spatial patterns change when *Chiropotes satanas chiropotes* inhabit forest fragments. *International Journal of Primatology*, **30**, 515-531.

Boyle, S.A., Smith, A.T. (2010) Can landscape and species characteristics predict primate presence in forest fragments in the Brazilian Amazon? *Biological Conservation*, **143**, 1134-1143.

Boyle, S.A., Smith, A.T. (2010) Behavioral modifications in northern bearded saki monkeys (*Chiropotes satanas chiropotes*) in forest fragments of central Amazonia. *Primates*, **51**, 43-51.

Camargo, C.C. & Ferrari, S.F. (2007) Interactions between tayras (*Eira barbara*) and red-handed howlers (*Alouatta belzebul*) in eastern Amazonia. *Primates*, **48**, 147-150.

Carvalho-Jr., O. (2003) Primates in a forest fragment in eastern Amazonia. *Neotropical Primates*, **11(2)**, 100-103.

Ferrari, S.F., Bobadilla, U.L. & Emidio-Silva, C. (2007) Where have all the titis gone? The heterogeneous distribution of *Callicebus moloch* in eastern Amazonia, and its implications for the conservation of Amazonian primates. *Primate Conservation*, **22**, 49-54.

Fialho, M.S. (2010) Contribuição à Distribuição do Gênero *Mico*, (Callitrichidae, Primates) no Médio Teles Pires, Jacareacanga, Pará. *Neotropical Primates*, **17(1)**, 31-32.

Gregorin, R. (2006) Taxonomia e variação geográfica das espécies do gênero *Alouatta* Lacépède (Primates, Atelidae) no Brasil. *Revista Brasileira de Zoologia*, **23(1)**, 64-144.

Haugaasen, T. & Peres, C.A. (2007) Vertebrate responses to fruit production in Amazonian flooded and unflooded forests. *Biodiversity and Conservation*, **16**, 4165-4190.

Haugaasen, T. & Peres, C.A. (2009) Interspecific primate associations in Amazonian flooded and unflooded forests. *Primates*, **50**, 239-251.

Menezes-Costa, A., Machado-Ferreira, E., Voloch, C.M., Bonvicino, C.R., Seuánez, H.N., Leoncini, O. & Soares, C.A.G. (2013) Identification of Bacterial Infection in Neotropical Primates. *Microbial* *Ecology*, **66**, 471-478.

Monteiro, F.O.B., Kugelmeier, T., Rio do Valle, R., Lima, A.B.F., Silva, F.E., Martins, S.S., Pereira, L.G., Dinucci, K.L. & Viau, P. (2013) Evaluation of the fecal steroid concentrations in *Alouatta belzebul* (Primates, Atelidae) in the National Forest of Tapirape-Aquiri in Pará, Brazil. **Journal of Medical Primatology**, **42**, 325-332.

Oliveira, A.C.M. & Ferrari, S.F. (2000) Seed dispersal by black-handed tamarins, *Saguinus midas niger* (Callitrichinae, Primates): implications for the regeneration of degraded forest habitats in eastern Amazonia. *Journal of Tropical Ecology*, **16**, 709-716.

Oliveira, A.C.M. & Ferrari, S.F. (2008) Habitat exploitation by free-ranging *Saguinus niger* in eastern Amazonia. *International Journal of Primatology*, **29**, 1499-1510.

Oliveira, M.L., Baccaro, F.B., Braga-Neto, R. & Magnusson, W.E. (2008) *Reserva Ducke: A biodiversidade amazônica através de uma grade.* Áttema Design Editorial, Manaus.

Oliveira, L.C., Loretto, D., Viana, L.R., Silva-Jr., J.S. & Fernandes, W.G. (2009) Primate community of the tropical rain forests of Saracá-Taqüera National Forest, Pará, Brazil. *Brazilian Journal of Biology*, **69(3)**, 631-637.

Parry, L., Barlow, J. & Peres, C.A. (2007) Large-vertebrate assemblages of primary and secondary forests in the Brazilian Amazon. *Journal of Tropical Ecology*, **23**, 653-662.

Pimenta, F.E. & Silva-Júnior, J.S. (2005) An update on the distribution of Primates of the Tapajós-Xingu interfluvium, Central Amazonia. *Neotropical Primates*, **13(2)**, 23-28.

Pinheiro, T., Ferrari, S.F. & Lopes, M.A. (2011) Polyspecific Associations Between Squirrel Monkeys (*Saimiri sciureus*) and Other Primates in Eastern Amazonia. *American Journal of Primatology*, **73**, 1145-1151.

Pinheiro, T., Ferrari, S.F. & Lopes, M.A. (2013) Activity budget, diet, and use of space by two groups of squirrel monkeys (*Saimiri sciureus*) in eastern Amazonia. *Primates*, **54**, 301-308.

Pinto, L.P. & Setz, E.Z.F. (2000) Sympatry and new locality for *Alouatta belzebul discolor* and *Alouatta seniculus* in the southern Amazon. *Neotropical Primates*, **8(4)**, 150-151.

Pinto, L.P. & Setz, E.Z.F. (2004) Diet of *Alouatta belzebul discolor* in an amazonian rain forest of northern Mato Grosso state, Brazil. *International Journal of Primatology*, **25(6)**, 1197-1211.

Quintino, E.P. & Bicca-Marques, J.C. (2013) Predation of *Alouatta puruensis* by *Boa constrictor*. *Primates*, **54**, 325-330.

Silva, C.R. (2007) Registro de alimentação noturna em macaco-prego (*Cebus apella*). *Neotropical Primates*, **14(2)**, 72-74.

Silva, S.S.B. & Ferrari, S.F. (2009) Behavior patterns of southern bearded sakis (*Chiropotes satanas*) in the fragmented landscape of eastern Brazilian Amazonia. *American Journal of Primatology*, **71**, 1-7.

Souza, L.L., Queiroz, H.L. & Ayres, J.M. (2004) The mottled-face tamarin, *Saguinus inustus*, in the Amanã Sustainable Development Reserve, Amazonas, Brazil. *Neotropical Primates*, **12(3)**, 121-122.

Stone, A.I., Lima, E.M., Aguiar, G.F.S., Camargo, C.C., Flores, T.A., Kelt, D.A., Marques-Aguiar, S.A., Queiroz, J.A.L., Ramos, R.M. & Silva Júnior, J.S. (2009) Non-volant mammalian diversity in fragments in extreme eastern Amazonia. *Biodiversity and Conservation*, **18**, 1685-1694.

Vaz, S.M. (2001) Primatas da região do rio Tapajós, Pará, Brasil. *Neotropical Primates*, **9(2)**, 54-57.

Vulinec, K., Lambert, J.E. & Mellow, D.J. (2006) Primate and dung beetle communities in secondary growth rain forests: implications for conservation of seed dispersal systems. *International Journal of Primatology*, **27(3)**, 855-879.

***Aotus***

Bicca-Marques, J.C. & Garber, P.A. (2004) Use of spatial, visual, and olfactory information during foraging in wild nocturnal and diurnal anthropoids: A field experiment comparing *Aotus*, *Callicebus*, and *Saguinus*. *American Journal of Primatology* **62**, 171-187.

Calouro, A.M. (2005) Análise do manejo florestal de “baixo impacto” e da caça de subsistência sobre uma comunidade de primatas na Floresta Estadual do Antimary (Acre, Brasil). Tese de doutorado, Programa de Pós-Graduação em Ecologia e Recursos Naturais, Universidade Federal de São Carlos.

Camargo, C.C. & Ferrari, S.F. (2007) Interactions between tayras (*Eira barbara*) and red-handed howlers (*Alouatta belzebul*) in eastern Amazonia. *Primates*, **48**, 147-150.

Dominy, N.J., Garber, P.A., Bicca-Marques, J.C. & Azevedo-Lopes, M.A. (2003) Do female tamarins use visual cues to detect fruit rewards more successfully than do males? *Animal Behaviour*, **66**, 829-837.

Ferrari, S.F., Cruz Neto, E.H., Iwanaga, S. & Corrêa, H.K.M. (1996) An unusual primate community at the Estação Ecológica Serra dos Três Irmãos, Rondônia, Brazil. *Neotropical Primates*, **4(2)**, 55-56.

Garber, P.A. & Leigh, S.R. (2001) Patterns of positional behavior in mixed-species troops of *Callimico goeldii*, *Saguinus labiatus*, and *Saguinus fuscicollis* in northwestern Brazil. *American Journal of Primatology*, **54**, 17-31.

Groves, C.P. (2005) Order Primates. *Mammal Species of the World: A Taxonomic and Geographic Reference, Vol. 1* (ed. by D.E. Wilson and D.M. Reeder), pp. 111–184. Johns Hopkins University Press, Baltimore.

Hershkovitz, P. (1983) Two new species of night monkeys, genus *Aotus* (Cebidae, Platyrrhini): a preliminary report on *Aotus* taxonomy. *American Journal of Primatology*, **4**, 209-243.

Kasecker, T.P. (2006) *Efeito da estrutura do habitat sobre a riqueza e composição de comunidades de primatas da RDS Piagaçu-Purus, Amazônia Central, Brasil*. Dissertação de mestrado, Programa de Pós-Graduação em Biologia Tropical e Recursos Naturais, Universidade Federal do Amazonas.

Levi, T. & Peres, C.A. (2013) Dispersal vacuum in the seedling recruitment of a primate-dispersed Amazonian tree. *Biological Conservation*, **163**, 99-106.

Lopes, M.A.O.A. &Rehg, J.A. (2003) Observations of *Callimico goeldii* with *Saguinus imperator* in the Serra do Divisor National Park, Acre, Brazil. *Neotropical Primates*, **11 (3)**, 181-183.

Menezes-Costa, A., Machado-Ferreira, E., Voloch, C.M., Bonvicino, C.R., Seuánez, H.N., Leoncini, O. & Soares, C.A.G. (2013) Identification of Bacterial Infection in Neotropical Primates. *Microbial* *Ecology*, **66**, 471-478.

Oliveira, A.C.M. & Ferrari, S.F. (2000) Seed dispersal by black-handed tamarins, *Saguinus midas niger* (Callitrichinae, Primates): implications for the regeneration of degraded forest habitats in eastern Amazonia. *Journal of Tropical Ecology*, **16**, 709-716.

Peres, C.A. (1988) Primate community structure in western Brazilian Amazonia. *Primate Conservation*, **9**, 83-86.

Pimenta, F.E. &Silva-Júnior, J.S. (2005) An update on the distribution of Primates of the Tapajós-Xingu interfluvium, Central Amazonia. *Neotropical Primates*, **13(2)**, 23-28.

Pinheiro, T., Ferrari, S.F. & Lopes, M.A. (2011) Polyspecific Associations Between Squirrel Monkeys (*Saimiri sciureus*) and Other Primates in Eastern Amazonia. *American Journal of Primatology*, **73**, 1145-1151.

Pinheiro, T., Ferrari, S.F. & Lopes, M.A. (2013) Activity budget, diet, and use of space by two groups of squirrel monkeys (*Saimiri sciureus*) in eastern Amazonia. *Primates*, **54**, 301-308.

Rehg, J.A. (2006) Seasonal variation in polyspecific associations among *Callimico goeldii*, *Saguinus labiatus*, and *S. fuscicollis* in Acre, Brazil. *International Journal of Primatology*, **27(5)**, 1399-1428.

Rylands, A.B., Schneider, H., Langguth, A., Mittermeier, R.A., Groves, C.P. & Rodríguez-Luna, E. (2000) An assessment of the diversity of New World primates. *Neotropical Primates*, **8(2)**, 61-93.

Rylands, A.B., Mittermeier, R.A. & Silva Jr., J.S. (2012) Neotropical primates: taxonomy and recently described species and subspecies. *International Zoo Yearbook*, **46**, 11-24.

Silva, S.S.B. & Ferrari, S.F. (2009) Behavior patterns of southern bearded sakis (*Chiropotes satanas*) in the fragmented landscape of eastern Brazilian Amazonia. *American Journal of Primatology*, **71**, 1-7.

Silva-Júnior, J.S., Nunes, A.& Fernandes, M.E.B. (1995) Geographic distribution of night monkeys, *Aotus*, in northern Brazil: new data and a correction. *Neotropical Primates*, **3(3)**, 72-74.

Silva-Júnior, J.S. & Fernandes, M.E.B. (1999) A northeastern extension of the distribution of *Aotus infulatus* in Maranhão, Brazil. *Neotropical Primates*, **7(3)**, 76-80.

Stone, A.I., Lima, E.M., Aguiar, G.F.S., Camargo, C.C., Flores, T.A., Kelt, D.A., Marques-Aguiar, S.A., Queiroz, J.A.L., Ramos, R.M. & Silva Júnior, J.S. (2009) Non-volant mammalian diversity in fragments in extreme eastern Amazonia. *Biodiversity and Conservation*, **18**, 1685-1694.

Vaz, S.M. (2001) Primatas da região do rio Tapajós, Pará, Brasil. *Neotropical Primates*, **9(2)**, 54-57.

***Ateles***

Boubli, J.P. (1999) Feeding ecology of black-headed uacaris (*Cacajao melanocephalus melanocephalus*) in Pico da Neblina National Park, Brazil. *International Journal of Primatology*, **20(5)**, 719-749.

Boyle, S.A., Smith, A.T. (2010) Can landscape and species characteristics predict primate presence in forest fragments in the Brazilian Amazon? *Biological Conservation*, **143**, 1134-1143.

Boyle, S.A., Smith, A.T. (2010) Behavioral modifications in northern bearded saki monkeys (*Chiropotes satanas chiropotes*) in forest fragments of central Amazonia. *Primates*, **51**, 43-51.

Cabral, M.M.M., Mattos, G.E. & Rosas, F.C.W. (2008) Mammals, birds and reptiles in Balbina reservoir, state of Amazonas, Brazil. *Check List*, **4(2)**, 152-158.

Fialho, M.S. (2010) Contribuição à Distribuição do Gênero *Mico*, (Callitrichidae, Primates) no Médio Teles Pires, Jacareacanga, Pará. *Neotropical Primates*, **17(1)**, 31-32.

Groves, C.P. (2005) Order Primates. *Mammal Species of the World: A Taxonomic and Geographic Reference, Vol. 1* (ed. by D.E. Wilson and D.M. Reeder), pp. 111–184. Johns Hopkins University Press, Baltimore.

Haugaasen, T. & Peres, C.A. (2005) Mammal assemblage structure in Amazonian flooded and unflooded forests. *Journal of Tropical Ecology*, **21**, 133-145.

Haugaasen, T. & Peres, C.A. (2007) Vertebrate responses to fruit production in Amazonian flooded and unflooded forests. *Biodiversity and Conservation*, **16**, 4165-4190.

Haugaasen, T. & Peres, C.A. (2009) Interspecific primate associations in Amazonian flooded and unflooded forests. *Primates*, **50**, 239-251.

Iwanaga, S. & Ferrari, S.F. (2002) Geographic distribution and abundance of woolly (*Lagothrix cana*) and spider (*Ateles chamek*) monkeys in southwestern brazilian Amazonia. *American Journal of Primatology*, **56**, 57-64.

Iwanaga, S. & Ferrari, S.F. (2002) Geographic distribution of red howlers (*Alouatta seniculus*) in southwestern Brazilian Amazonia, with notes on *Alouatta caraya*. *International Journal of Primatology*, **23(6)**, 1245-1256.

Kellogg, R. & Goldman, E.A. (1944) Review of the spider monkeys. *Proceedings of the United States National Museum*, **96**, 1-45.

Levi, T. & Peres, C.A. (2013) Dispersal vacuum in the seedling recruitment of a primate-dispersed Amazonian tree. *Biological Conservation*, **163**, 99-106.

Lopes, M.A.O.A. & Rehg, J.A. (2003) Observations of *Callimico goeldii* with *Saguinus imperator* in the Serra do Divisor National Park, Acre, Brazil. *Neotropical Primates*, **11(3)**, 181-183.

Mourthé, I. (2011) Reactions of White-Bellied Spider Monkeys to a Predation Attempt by a Cougar. *Neotropical Primates*, **18(1)**, 28-29.

Oliveira, L.C., Loretto, D., Viana, L.R., Silva-Jr., J.S. & Fernandes, W.G. (2009) Primate community of the tropical rain forests of Saracá-Taqüera National Forest, Pará, Brazil. *Brazilian Journal of Biology*, **69(3)**, 631-637.

Oliveira, M.L., Baccaro, F.B., Braga-Neto, R. & Magnusson, W.E. (2008) *Reserva Ducke: A biodiversidade amazônica através de uma grade*. Áttema Design Editorial, Manaus.

Parry, L., Barlow, J. & Peres, C.A. (2007) Large-vertebrate assemblages of primary and secondary forests in the Brazilian Amazon. *Journal of Tropical Ecology*, **23**, 653-662.

Parry, L., Barlow, J. & Peres, C.A. (2009) Allocation of hunting effort by Amazonian smallholders: Implications for conserving wildlife in mixed-use landscapes. *Biological Conservation*, **142**, 1777-1786.

Peres, C.A. (1988) Primate community structure in western Brazilian Amazonia. *Primate Conservation*, **9**, 83-86.

Pimenta, F.E. & Silva-Júnior, J.S. (2005) An update on the distribution of Primates of the Tapajós-Xingu interfluvium, Central Amazonia. *Neotropical Primates*, **13(2)**, 23-28.

Pontes, A.R.M. (1999) Environmental determinants of primate abundance in Maracá Island, Roraima, Brazilian Amazonia. *Journal of Zoology*, **247**, 189-199.

Ravetta, A.L. & Ferrari, S.F. (2009) Geographic distribution and population characteristics of the endangered white-fronted spider monkey (*Ateles marginatus*) on the lower Tapajós River in central Brazilian Amazonia. *Primates*, **50(3)**, 261-268.

Rylands, A.B., Schneider, H., Langguth, A., Mittermeier, R.A., Groves, C.P. & Rodríguez-Luna, E. (2000) An assessment of the diversity of New World primates. *Neotropical Primates*, **8(2)**, 61-93.

Rylands, A.B., Mittermeier, R.A. & Silva Jr., J.S. (2012) Neotropical primates: taxonomy and recently described species and subspecies. *International Zoo Yearbook*, **46**, 11-24.

Rylands, A.B. & Mittermeier, R.A. (2009) The diversity of the New World primates (Platyrrhini). *South American primates: comparative perspectives in the study of bahavior, ecology, and conservation* (ed. by P.A. Garber, A. Estrada, J.C. Bicca-Marques, E.W. Heymann and K.B. Strier), pp. 23–54. Springer, New York.

Silva, C.R. (2007) Registro de alimentação noturna em macaco-prego (*Cebus apella*). *Neotropical Primates*, **14(2)**, 72-74.

Vaz, S.M. (2001) Primatas da região do rio Tapajós, Pará, Brasil. *Neotropical Primates*, **9(2)**, 54-57.

***Cacajao***

Barnett, A.A., Castilho, C.V., Shapley, R.L. & Anicácio, A. (2005) Diet, habitat selection and natural history of *Cacajao melanocephalus ouakary* in Jaú National Park, Brazil. *International Journal of Primatology*, **26(4)**, 949-969.

Barnett, A.A., Schiel, V., Deveny, A., Valsko, J., Spironello, W.R. & Ross, C. (2011) Predation on *Cacajao ouakary* and *Cebus albifrons* (Primates: Platyrrhini) by harpy eagles. *Mammalia*, **75**, 169-172.

Barnett, A.A., Almeida, T., Spironello, W.R., Silva, W.S., MacLarnon, A. & Ross, C. (2012) Terrestrial foraging by *Cacajao melanocephalus ouakary* (Primates) in Amazonian Brazil: is choice of seed patch size and position related to predation risk? Folia Primatologica, 83, 126-139.

Barnett, A.A., Boyle, S.A., Pinto, L.P., Lourenço, W.C., Almeida, T., Silva, W.S., Ronchi-Teles, B., Bezerra, B.M. Ross, C., MacLarnon, A. & Spironello, W.R. (2012) Primary seed dispersal by three Neotropical seed-predating primates (*Cacajao melanocephalus ouakary, Chiropotes chiropotes* and *Chiropotes albinasus*). *Journal of Tropical Ecology*, **28(06)**, 543-555.

Barnett, A.A., Shaw, P., Spironello, W.R., MacLarnon, A. & Ross, C.(2012) Sleeping site selection by golden-backed uacaris, *Cacajao melanocephalus ouakary* (Pitheciidae), in Amazonian flooded forests. *Primates*, **53**, 273-285.

Barnett, A.A., Boyle, S.A., Norconk, M.M., Palminteri, S., Santos, R.R., Veiga, L.M. Alvim, T.H.G., Bowler, M., Chism, J., Di Fiore, A., Fernandez-Duque, E., Guimarães, A.C.P., Harrison-Levine, A., Haugaasen, T., Lehman, S., Mackinnon, K.C., Melo, F.R., Moreira, L.S., Moura, V.S., Phillips, C.R., Pinto, L.P., Port-Carvalho, M., Setz, E.Z.F., Shaffer, C., Silva, L.R., Silva, S.S.B., Soares, R.F., Thompson, C.L., Vieira, T.M., Vreedzaam, A., Walker-Pacheco, S.E., Spironello, W.R., Maclarnon, A. & Ferrari, S.F. Terrestrial Activity In Pitheciins (Cacajao, Chiropotes, And Pithecia). *American Journal of Primatology* **74**: 1106-1127.

Barnett, A.A., Ronchi-Teles, B., Almeida, T., Deveny, A., Schiel-Baracuhy, V., Souza-Silva, W., Spironello, W., Ross, C. & MacLarnon, A. (2013) Arthropod predation by a specialist seed predator, the Golden-backed Uacari (Cacajao melanocephalus ouakary, Pitheciidae) in Brazilian Amazonia. International *Journal of Primatology* **34**: 470-485.

Bezerra, B.M., Souto, A. & Jones, G. (2010) Vocal Repertoire of Golden-backed Uakaris (*Cacajao melanocephalus*): Call Structure and Context. *International Journal of Primatology*, **31**, 759-778.

Bezerra, B.M., Souto, A. & Jones, G. (2010) Responses of golden-backed uakaris, *Cacajao melanocephalus*, to call playback: implications for surveys in the flooded Igapó forest. *Primates*, **51**, 327-336.

Bezerra, B.M., Barnett, A.A., Souto, A. & Jones, G. (2011) Ethogram and natural history of Golden-backed Uakaris (*Cacajao melanocephalus*). *International Journal of Primatology*, **32**, 46-68.

Bezerra, B.M., Souto, A.S. & Jones, G. (2012) Propagation of the loud ‘‘tchó’’ call of golden-backed uakaris, *Cacajao melanocephalus*, in the black-swamp forests of the upper Amazon. *Primates,* **53**, 317-325.

Boubli, J.P. (2002) Western extension of the range of bearded sakis: a possible new taxon of *Chiropotes* sympatric with *Cacajao* in the Pico da Neblina National Park, Brazil. *Neotropical Primates*, **10(1)**, 1-4.

Boubli, J.P., Silva, M.N.F., Amado, M.V., Hrbek, T., Pontual, F.B. & Farias, I.P. (2008) A taxonomic reassessment of *Cacajao melanocephalus* Humbolt (1811), with the description of two new species. *International Journal of Primatology*, **29**, 723-741.

Bowler, M., Knogge, C., Heymann, E.W. & Zinner, D. (2012) Multilevel Societies in New World Primates? Flexibility May Characterize the Organization of Peruvian Red Uakaris (*Cacajao calvus ucayalii*). *International Journal of Primatology*, **33**, 1110-1124.

Lopes, M.A.O.A. & Rehg, J.A. (2003) Observations of *Callimico goeldii* with *Saguinus imperator* in the Serra do Divisor National Park, Acre, Brazil. *Neotropical Primates*, **11(3)**, 181-183.

Peres, C.A. (1988) Primate community structure in western Brazilian Amazonia. *Primate Conservation*, **9**, 83-86.

Silva-Júnior, J.A. & Martins, E.S. (1999) On a new White bald uakari population in southwestern Brazilian Amazonia. *Neotropical Primates*, **7(4)**, 119-121.

Souza, L.L., Queiroz, H.L. & Ayres, J.M. (2004) The mottled-face tamarin, *Saguinus inustus*, in the Amanã Sustainable Development Reserve, Amazonas, Brazil. *Neotropical Primates*, **12(3)**, 121-122.

***Callibella***

Garbino, G.S.T., Silva, F.E. & Davis, B.J.W. (2013) Range extension of the vulnerable dwarf marmoset, *Callibella humilis* (Roosmalen et al. 1998), and first analysis of its long call structure. *Primates*, **54**, 331-334.

van Roosmalen, M.G.M. & van Roosmalen, T. (2003) The description of a new marmoset genus, *Callibella* (Callitrichinae, Primates), including its molecular phylogenetic status. *Neotropical Primates*, **11(1)**, 1-10.

***Callicebus***

Auricchio, P. (2010) A morphological analysis of some species of *Callicebus*, Thomas, 1903 (Pitheciidae - Callicebinae). *Neotropical Primates*, **17(2)**, 47-58.

Barlow, J. & Peres, C.P. (2006) Effects of single and recurrent wildfires on fruit production and large vertebrate abundance in a central Amazonian forest. *Biodiversity and Conservation*, **15**, 985-1012.

Bezerra, B.M., Barnett, A.A., Souto, A. & Jones, G. (2011) Ethogram and natural history of Golden-backed Uakaris (*Cacajao melanocephalus*). *International Journal of Primatology*, **32**, 46-68.

Bonvicino, C.R., Penna-Firme, V., Nascimento, F.F., Lemos, B., Stanyon, R. & Seuánez, H.N. (2003) The lowest diploid number (2n = 16) yet found in any primate: *Callicebus lugens* (Humboldt, 1811). *Folia Primatologica*, **74**, 141-149.

Camargo, C.C. & Ferrari, S.F. (2007) Interactions between tayras (*Eira barbara*) and red-handed howlers (*Alouatta belzebul*) in eastern Amazonia. *Primates*, **48**, 147-150.

Casado, F., Bonvicino, C.R. & Seuánez, H.N. (2007) Phylogeographic analyses of *Callicebus lugens* (Platyrrhini, Primates). *Journal of Heredity*, **98(1)**, 88-92.

Gualda-Barros, J., Nascimento, F.O. & Amaral, M.K. (2012) A new species of *Callicebus* Thomas, 1903 (Primat es, Pitheciidae) from the states of Mato Grosso and Pará, Brazil. *Papéis Avulsos de Zoologia*, **52(23)**, 261-279.

Groves, C.P. (2005) Order Primates. *Mammal species of the world: A taxonomic and geographic reference* (ed. by D.E. Wilson and D.M. Reeder), pp. 111–184. Johns Hopkins University Press, Baltimore.

Haugaasen, T. & Peres, C.A. (2007) Vertebrate responses to fruit production in Amazonian flooded and unflooded forests. *Biodiversity and Conservation*, **16**, 4165-4190.

Haugaasen, T. & Peres, C.A. (2009) Interspecific primate associations in Amazonian flooded and unflooded forests. *Primates*, **50**, 239-251.

Kasecker, T.P. (2006) *Efeito da estrutura do habitat sobre a riqueza e composição de comunidades de primatas da RDS Piagaçu-Purus, Amazônia Central, Brasil*. Dissertação de mestrado, Programa de Pós-Graduação em Biologia Tropical e Recursos Naturais, Universidade Federal do Amazonas.

Lees, A.C. & Peres, C.A. (2008) Conservation value of remnant riparian forest corridors of varying quality for amazonian birds and mammals. *Conservation Biology*, **22(2)**, 439-449.

Levi, T. & Peres, C.A. (2013) Dispersal vacuum in the seedling recruitment of a primate-dispersed Amazonian tree. *Biological Conservation*, **163**, 99-106.

Menescal, L.A., Gonçalves, E.C., Silva, A., Ferrari, S.F. & Schneider, M.P.C. (2009) Genetic diversity of red-bellied titis (*Callicebus moloch*) from eastern Amazonia based on microsatellite markers. *Biochemical Genetics*, **47**, 235-240.

Michalski, F.& Peres, C.A. (2005) Anthropogenic determinants of primate and carnivore local extinctions in a fragmented forest landscape of southern Amazonia. *Biological Conservation*, **124**, 383-396.

Michalski, F. & Peres, C.A. (2007) Disturbance-mediated mammal persistence and abundance-area relationships in Amazonian forest fragments. *Conservation Biology*, **21(6)**, 1626-1640.

Monção, G.R., Selhorst, V. & Soares-Filho, J.A.R. (2008) Expansão da distribuição geográfica de *Callicebus bernhardi* a oeste do Rio Ji-Paraná, Estado de Rondônia, Brasil. *Neotropical Primates*, **15(2)**, 67-68.

Noronha, M.A., Spironello, W.R. & Ferreira, D.C. (2007) New occurrence records and eastern extension to the range of *Callicebus cinerascens* (Primates, Pitheciidae). *Neotropical Primates*, **14(3)**, 137-139.

Pimenta, F.E. & Silva-Júnior, J.S. (2005) An update on the distribution of primates of the Tapajós-Xingu interfluvium, Central Amazonia. *Neotropical Primates*, **13(2)**, 23-28.

Pinheiro, T., Ferrari, S.F. & Lopes, M.A. (2011) Polyspecific Associations Between Squirrel Monkeys (*Saimiri sciureus*) and Other Primates in Eastern Amazonia. *American Journal of Primatology*, **73**, 1145-1151.

Pinheiro, T., Ferrari, S.F. & Lopes, M.A. (2013) Activity budget, diet, and use of space by two groups of squirrel monkeys (*Saimiri sciureus*) in eastern Amazonia. *Primates*, **54**, 301-308.

Pinto, L.P. & Setz, E.Z.F. (2004) Diet of *Alouatta belzebul discolor* in an Amazonian rain forest of northern Mato Grosso State, Brazil. *International Journal of Primatology,* **25(6)**, 1197-1211.

Röhe, F. & Silva-Júnior, J.S. (2009) Confirmation of *Callicebus dubius* (Pitheciidae) Distribution and Evidence of Invasion into the Geographic Range of *Callicebus stephennashi*. *Neotropical Primates*, **16(2)**, 71-73.

Silva, F.H.A. (2007) *Dieta do gavião-real Harpia harpyja (Aves: Accipitridae) em florestas de terra firme de Parintins, Amazonas, Brasil.* Dissertação de mestrado, Programa de Pós-Graduação em Biologia Tropical e Recursos Naturais, Universidade Federal do Amazonas.

Silva-Júnior, J.S., Figueiredo-Ready, W.M.B. & Ferrari, S.F. (2013) Taxonomy and geographic distribution of the Pitheciidae. *Evolutionary biology and conservation of Titis, Sakis and Uacaris* (ed. by L.M. Veiga, A.A. Barnett, S.F. Ferrari and M.A. Norconk), pp. 31-42. Cambridge University Press, Cambridge.

Souza, L.L., Queiroz, H.L. & Ayres, J.M. (2004) The mottled-face tamarin, *Saguinus inustus*, in the Amanã Sustainable Development Reserve, Amazonas, Brazil. *Neotropical Primates*, **12(3)**, 121-122.

van Roosmalen, M.G.M., van Roosmalen, T. & Mittermeier, R.A. (2002) A taxonomic review of the titi monkeys, genus *Callicebus* Thomas, 1903, with the description of two new species, *Callicebus bernhardi* and *Callicebus stephennashi*, from brazilian Amazonia. *Neotropical Primates*, **10 (Suppl.)**, 1-52.

Vaz, S.M. (2001) Primatas da região do Rio Tapajós, Pará, Brasil. *Neotropical Primates*, **9(2)**, 54-57.

***Callimico***

Botelho, A.L.M., Calouro, A.M., Borges, L.H.M. & Chaves, W.A. (2012) Large and medium-sized mammals of the Humaitá Forest Reserve, southwestern Amazonia, state of Acre, Brazil. *Checklist*, **8(6)**, 1190-1195.

Calouro, A.M. (2005) *Análise do manejo florestal de “baixo impacto” e da caça de subsistência sobre uma comunidade de primatas na Floresta Estadual do Antimary (Acre, Brasil)*. Tese de doutorado, Programa de Pós-Graduação em Ecologia e Recursos Naturais, Universidade Federal de São Carlos.

Garber, P.A. & Leigh, S.R. (2001) Patterns of positional behavior in mixed-species troops of *Callimico goeldii*, *Saguinus labiatus*, and *Saguinus fuscicollis* in northwestern Brazil. *American Journal of Primatology*, **54**, 17-31.

Hershkovitz, P. (1977) *Living new world monkeys (Platyrrhini), Vol. 1*. The university of Chicago Press, Chicago.

Lopes, M.A.O.A. & Rehg, J.A. (2003) Observations of *Callimico goeldii* with *Saguinus imperator* in the Serra do Divisor National Park, Acre, Brazil. *Neotropical Primates*, **11(3)**, 181-183.

Rehg, J.A. (2006) Seasonal variation in polyspecific associations among *Callimico goeldii*, *Saguinus labiatus*, and *S. fuscicollis* in Acre, Brazil. *International Journal of Primatology*, **27(5)**, 1399-1428.

Rehg, J.A. (2010) Plant Feeding Patches: Patterns of Use by Associating *Callimico goeldii*, *Saguinus labiatus*, and *S. fuscicollis*. *Neotropical Primates*, **17(1)**, 18-21.

***Cebuella***

Bicca-Marques, J.C. & Calegaro-Marques, C. (1995) Updating the known distribution of the pygmy marmoset (*Cebuella pygmaea*) in the state of Acre, Brazil. *Neotropical Primates*, **3(2)**, 48-49.

Calouro, A.M. (2005) *Análise do manejo florestal de “baixo impacto” e da caça de subsistência sobre uma comunidade de primatas na Floresta Estadual do Antimary (Acre, Brasil)*. Tese de doutorado, Programa de Pós-Graduação em Ecologia e Recursos Naturais, Universidade Federal de São Carlos.

Canizo, R.O.A. & Calouro, A.A. (2011) Observação de Comportamento Agonístico de *Cebuella pygmaea* Sobre *Sciurus spadiceus* em um Fragmento Florestal No Estado do Acre, Brasil. *Neotropical Primates*, **18(2)**, 60-62.

Dominy, N.J., Garber, P.A., Bicca-Marques, J.C. & Azevedo-Lopes, M.A. (2003) Do female tamarins use visual cues to detect fruit rewards more successfully than do males? *Animal Behaviour*, **66**, 829-837.

Haugaasen, T. & Peres, C.A. (2005) Mammal assemblage structure in Amazonian flooded and unflooded forests. *Journal of Tropical Ecology*, **21**, 133-145.

Hershkovitz, P. (1977) *Living new world monkeys (Platyrrhini), Vol. 1.* The University of Chicago Press, Chicago.

Messias, M.R., Coragem, J.T., Gomes, I.S.R., Oliveira, M.A., Bonavigo, P.H., Nienow, S.S. & Souza, E.S. (2011) Southern Extension of the Geographical Range of the Pygmy Marmoset *Cebuella pygmaea niveiventris* (Lönnberg, 1940) in the Southwestern Amazon Basin, State of Rondônia, Brazil. *Neotropical Primates*, **18(1)**, 30-31.

Peres, C.A. (1988) Primate community structure in western Brazilian Amazonia. *Primate Conservation*, **9**, 83-86.

Santos, F.G.A., Bicca-Marques, J.C., Calegaro-Marques, C., Farias, E.M.P. & Azevedo, M.A.O. (1995) On the occurrence of parasites in free-ranging callitrichids. *Neotropical Primates*, **3(2)**, 46-47.

van Roosmalen, M.G.M. & van Roosmalen, T. (1997) An eastern extension of the geographical range of the pigmy marmoset, *Cebuella pygmaea*. *Neotropical Primates*, **5(1)**, 3-6.

***Cebus* and *Sapajus***

Alfaro, J.W.L., Silva Jr., J.S. & Rylands, A.B. (2012) How different are robust and gracile capuchin monkeys? An argument for the use of *Sapajus* and *Cebus*. *American Journal of Primatology*, **74**, 273-286.

Barlow, J. & Peres, C.P. (2006) Effects of single and recurrent wildfires on fruit production and large vertebrate abundance in a central Amazonian forest. *Biodiversity and Conservation*, **15**, 985-1012.

Barnett, A.A., Schiel, V., Deveny, A., Valsko, J., Spironello, W.R. & Ross, C. (2011) Predation on *Cacajao ouakary* and *Cebus albifrons* (Primates: Platyrrhini) by harpy eagles. *Mammalia*, **75**, 169-172.

Benchimol, M. & Venticinque, E.M. (2010) Harpy eagle (*Harpia harpyja*) predation on an infant brown capuchin monkey (*Cebus apella*) in the Brazilian Amazon. *Revista Brasileira de Ornitologia*, **18(4)**, 352-354.

Bobadilla, U.L. & Ferrari, S.F. (2000) Habitat use by *Chiropotes satanas utahicki* and syntopic Platyrrhines in eastern Amazonia. *American Journal of Primatology*, **50**, 215-224.

Botelho, A.L.M., Calouro, A.M., Borges, L.H.M. & Chaves, W.A. (2012) Large and medium-sized mammals of the Humaitá Forest Reserve, southwestern Amazonia, state of Acre, Brazil. *Checklist*, **8(6)**, 1190-1195.

Boubli, J.P. (1999) Feeding ecology of black-headed uacaris (*Cacajao melanocephalus melanocephalus*) in Pico da Neblina National Park, Brazil. *International Journal of Primatology*, **20(5)**, 719-749.

Boyle, S.A., Lourenço, W.C., Silva, L.R. & Smith, A.T. (2009) Travel and spatial patterns change when *Chiropotes satanas chiropotes* inhabit forest fragments. *International Journal of Primatology* **30**, 515-531.

Boyle, S.A., Smith, A.T. (2010) Can landscape and species characteristics predict primate presence in forest fragments in the Brazilian Amazon? *Biological Conservation*, **143**, 1134-1143.

Boyle, S.A., Smith, A.T. (2010) Behavioral modifications in northern bearded saki monkeys (*Chiropotes satanas chiropotes*) in forest fragments of central Amazonia. *Primates*, **51**, 43-51.

Cabral, M.M.M., Mattos, G.E. & Rosas, F.C.W. (2008) Mammals, birds and reptiles in Balbina reservoir, state of Amazonas, Brazil. *Check List*, **4(2)**, 152-158.

Calouro, A.M. (2005) *Análise do manejo florestal de “baixo impacto” e da caça de subsistência sobre uma comunidade de primatas na Floresta Estadual do Antimary (Acre, Brasil)*. Tese de doutorado, Programa de Pós-Graduação em Ecologia e Recursos Naturais, Universidade Federal de São Carlos.

Camargo, C.C. & Ferrari, S.F. (2007) Interactions between tayras (*Eira barbara*) and red-handed howlers (*Alouatta belzebul*) in eastern Amazonia. *Primates*, **48**, 147-150.

Carvalho Jr., O., Pinto, A.C.B. & Galetti, M. (1999) New observation on *Cebus kaapori* Queiroz, 1992, in eastern Brazilian Amazonia. *Neotropical Primates*, **7(2)**, 41-43.

Ferrari, S.F., Cruz Neto, E.H., Iwanaga, S. & Corrêa, H.K.M. (1996) An unusual primate community at the Estação Ecológica Serra dos Três Irmãos, Rondônia, Brazil. *Neotropical Primates*, **4(2)**, 55-56.

Ferrari, S.F., Bobadilla, U.L. & Emidio-Silva, C. (2007) Where have all the titis gone? The heterogeneous distribution of *Callicebus moloch* in eastern Amazonia, and its implications for the conservation of Amazonian primates. *Primate Conservation*, **22**, 49-54.

Fialho, M.S. (2010) Contribuição à Distribuição do Gênero *Mico*, (Callitrichidae, Primates) no Médio Teles Pires, Jacareacanga, Pará. *Neotropical Primates*, **17(1)**, 31-32.

Garber, P.A. & Leigh, S.R. (2001) Patterns of positional behavior in mixed-species troops of *Callimico goeldii*, *Saguinus labiatus*, and *Saguinus fuscicollis* in northwestern Brazil. *American Journal of Primatology*, **54**, 17-31.

Gumier-Costa, F. & Sperber, C.F. (2009) Atropelamentos de vertebrados na Floresta Nacional de Carajás, Pará, Brasil. *Acta Amazonica*, **39(2)**, 459-466.

Haugaasen, T. & Peres, C.A. (2005) Mammal assemblage structure in Amazonian flooded and unflooded forests. *Journal of Tropical Ecology*, **21**, 133-145.

Haugaasen, T. & Peres, C.A. (2007) Vertebrate responses to fruit production in Amazonian flooded and unflooded forests. *Biodiversity and Conservation*, **16**, 4165-4190.

Haugaasen, T. & Peres, C.A. (2009) Interspecific primate associations in Amazonian flooded and unflooded forests. *Primates*, 50, 239-251.

Kasecker, T.P. (2006) *Efeito da estrutura do habitat sobre a riqueza e composição de comunidades de primatas da RDS Piagaçu-Purus, Amazônia Central, Brasil.* Dissertação de mestrado, Programa de Pós-Graduação em Biologia Tropical e Recursos Naturais, Universidade Federal do Amazonas.

Lees, A.C. & Peres, C.A. (2008) Conservation value of remnant riparian forest corridors of varying quality for amazonian birds and mammals. *Conservation Biology*, **22(2)**, 439-449.

Levi, T. & Peres, C.A. (2013) Dispersal vacuum in the seedling recruitment of a primate-dispersed Amazonian tree. *Biological Conservation*, **163**, 99-106.

Lopes, M.A.O.A. & Rehg, J.A. (2003) Observations of *Callimico goeldii* with *Saguinus imperator* in the Serra do Divisor National Park, Acre, Brazil. *Neotropical Primates,* **11(3)**, 181-183.

Martins, S.S., Lima, E.M. & Silva-Júnior, J.S. (2005) Predation of a bearded saki (*Chiropotes utahicki*) by a harpy eagle (*Harpia harpyja*). *Neotropical Primates*, **13(1)**, 7-10.

Michalski, F. & Peres, C.A. (2005) Anthropogenic determinants of primate and carnivore local extinctions in a fragmented forest landscape of southern Amazonia. *Biological Conservation*, **124**, 383-396.

Michalski, F. & Peres, C.A. (2007) Disturbance-mediated mammal persistence and abundance-area relationships in Amazonian forest fragments. *Conservation Biology*, **21(6)**, 1626-1640.

Oliveira, A.C.M. & Ferrari, S.F. (2000) Seed dispersal by black-handed tamarins, *Saguinus midas niger* (Callitrichinae, Primates): implications for the regeneration of degraded forest habitats in eastern Amazonia. *Journal of Tropical Ecology*, **16**, 709-716.

Oliveira, A.C.M. & Ferrari, S.F. (2008) Habitat exploitation by free-ranging *Saguinus niger* in eastern Amazonia. *International Journal of Primatology*, **29**, 1499-1510.

Oliveira, L.C., Loretto, D., Viana, L.R., Silva-Jr., J.S. & Fernandes, W.G. (2009) Primate community of the tropical rain forests of Saracá-Taqüera National Forest, Pará, Brazil. *Brazilian Journal of Biology*, **69(3)**, 631-637.

Oliveira, M.L., Baccaro, F.B., Braga-Neto, R. & Magnusson, W.E. (2008) *Reserva Ducke: A biodiversidade amazônica através de uma grade*. Áttema Design Editorial, Manaus.

Parry, L., Barlow, J. & Peres, C.A. (2007) Large-vertebrate assemblages of primary and secondary forests in the Brazilian Amazon. *Journal of Tropical Ecology*, **23**, 653-662.

Parry, L., Barlow, J. & Peres, C.A. (2009) Allocation of hunting effort by Amazonian smallholders: Implications for conserving wildlife in mixed-use landscapes. *Biological Conservation*, **142**, 1777-1786.

Peres, C.A. (1988) Primate community structure in western Brazilian Amazonia. *Primate Conservation*, **9**, 83-86.

Peres, C.A. & Nascimento, H.S. (2006) Impact of game hunting by the Kayapó of south-eastern Amazonia: implications for wildlife conservation in tropical forest indigenous reserves. *Biodiversity and Conservation*, **15**, 2627-2653.

Pimenta, F.E. & Silva-Júnior, J.S. (2005) An update on the distribution of Primates of the Tapajós-Xingu interfluvium, Central Amazonia. *Neotropical Primates*, **13(2)**, 23-28.

Pinheiro, T., Ferrari, S.F. & Lopes, M.A. (2011) Polyspecific Associations Between Squirrel Monkeys (*Saimiri sciureus*) and Other Primates in Eastern Amazonia. *American Journal of Primatology*, **73**, 1145-1151.

Pinheiro, T., Ferrari, S.F. & Lopes, M.A. (2013) Activity budget, diet, and use of space by two groups of squirrel monkeys (*Saimiri sciureus*) in eastern Amazonia. *Primates*, **54**, 301-308.

Pinto, L.P. & Setz, E.Z.F. (2004) Diet of *Alouatta belzebul discolor* in an amazonian rain forest of northern Mato Grosso state, Brazil. *International Journal of Primatology,* **25(6)**, 1197-1211.

Pontes, A.R.M. (1999) Environmental determinants of primate abundance in Maracá Island, Roraima, Brazilian Amazonia. *Journal of Zoology*, **247**, 189-199.

Rehg, J.A. (2006) Seasonal variation in polyspecific associations among *Callimico goeldii*, *Saguinus labiatus*, and *S. fuscicollis* in Acre, Brazil. *International Journal of Primatology*, **27(5)**, 1399-1428.

Rodrigues, L.F. & Vidal, M.D. (2011) Densidade e Tamanho Populacional de Primatas em uma Área de Terra Firme na Amazônia Central. *Neotropical Primates*, **18(1)**, 9-16.

Silva, C.R. (2007) Registro de alimentação noturna em macaco-prego (*Cebus apella*). *Neotropical Primates*, **14(2)**, 72-74.

Silva, F.H.A. (2007) *Dieta do gavião-real Harpia harpyja (Aves: Accipitridae) em florestas de terra firme de Parintins, Amazonas, Brasil*. Dissertação de mestrado, Programa de Pós-Graduação em Biologia Tropical e Recursos Naturais, Universidade Federal do Amazonas.

Silva, S.S.B. & Ferrari, S.F. (2009) Behavior patterns of southern bearded sakis (*Chiropotes satanas*) in the fragmented landscape of eastern Brazilian Amazonia. *American Journal of Primatology*, **71**, 1-7.

Silva-Júnior, J.S. (2001) *Especiação nos macacos-prego e caiaras, gênero Cebus Erxleben, 1777 (Primates, Cebidae)*. Tese de doutorado, Programa de Pós-Graduação em Genética, Universidade Federal do Rio de Janeiro.

Silva-Júnior, J.S. & Cerqueira, R. (1998) New data and a historical sketch on the geographical distribution of the ka’apor capuchin, *Cebus kaapori*, Queiroz, 1992. *Neotropical Primates* **6(4)**, 118-121.

Souza, L.L., Queiroz, H.L. & Ayres, J.M. (2004). The mottled-face tamarin, *Saguinus inustus*, in the Amanã Sustainable Development Reserve, Amazonas, Brazil. *Neotropical Primates* **12(3)**, 121-122.

Stone, A.I., Lima, E.M., Aguiar, G.F.S., Camargo, C.C., Flores, T.A., Kelt, D.A., Marques-Aguiar, S.A., Queiroz, J.A.L., Ramos, R.M. & Silva Júnior, J.S. (2009) Non-volant mammalian diversity in fragments in extreme eastern Amazonia. *Biodiversity and Conservation*, **18**, 1685-1694.

Vaz, S.M. (2001) Primatas da região do rio Tapajós, Pará, Brasil. *Neotropical Primates*, **9(2)**, 54-57.

Vulinec, K., Lambert, J.E. & Mellow, D.J. (2006) Primate and dung beetle communities in secondary growth rain forests: implications for conservation of seed dispersal systems. *International Journal of Primatology*, **27(3)**, 855-879.

Zimmerman, B., Peres, C.A., Malcolm, J.R. & Turner, T. (2001) Conservation and development alliances with the Kayapó of south-eastern Amazonia, a tropical forest indigenous people. *Environmental Conservation*, **28(1)**, 10-22.

***Chiropotes***

Barlow, J. & Peres, C.P. (2006) Effects of single and recurrent wildfires on fruit production and large vertebrate abundance in a central Amazonian forest. *Biodiversity and Conservation*, **15**, 985-1012.

Barnett, A.A., Boyle, S.A., Norconk, M.M., Palminteri, S., Santos, R.R., Veiga, L.M. Alvim, T.H.G., Bowler, M., Chism, J., Di Fiore, A., Fernandez-Duque, E., Guimarães, A.C.P., Harrison-Levine, A., Haugaasen, T., Lehman, S., Mackinnon, K.C., Melo, F.R., Moreira, L.S., Moura, V.S., Phillips, C.R., Pinto, L.P., Port-Carvalho, M., Setz, E.Z.F., Shaffer, C., Silva, L.R., Silva, S.S.B., Soares, R.F., Thompson, C.L., Vieira, T.M., Vreedzaam, A., Walker-Pacheco, S.E., Spironello, W.R., Maclarnon, A. & Ferrari, S.F. Terrestrial Activity In Pitheciins (Cacajao, Chiropotes, And Pithecia). *American Journal of Primatology* **74**: 1106-1127.

Barnett, A.A., Boyle, S.A., Pinto, L.P., Lourenço, W.C., Almeida, T., Silva, W.S., Ronchi-Teles, B., Bezerra, B.M. Ross, C., MacLarnon, A. & Spironello, W.R. (2012) Primary seed dispersal by three Neotropical seed-predating primates (*Cacajao melanocephalus ouakary, Chiropotes chiropotes and Chiropotes albinasus*). *Journal of Tropical Ecology*, **28(06)**, 543-555.

Bobadilla, U.L. & Ferrari, S.F. (1998) First detailed field data on *Chiropotes satanas* *utahicki* Hershkovitz, 1985. *Neotropical Primates*, **6(1)**, 17-18.

Bobadilla, U.L. & Ferrari, S.F. (2000) Habitat use by *Chiropotes satanas utahicki* and syntopic Platyrrhines in eastern Amazonia. *American Journal of Primatology*, **50**, 215-224.

Bonvicino, C.R., Boubli, J.P., Otazú, I.B., Almeida, F.C., Nascimento, F.F., Coura, J.R. & Seuánez, H.N. (2003) Morphologic, karyotypic, and molecular evidence of a new form of *Chiropotes* (Primates, Pitheciinae). *American Journal of Primatology*, **61**, 123-133.

Boubli, J.P. (2002) Western extension of the range of bearded sakis: a possible new taxon of *Chiropotes* sympatric with *Cacajao* in the Pico da Neblina National Park, Brazil. *Neotropical Primates*, **10(1)**, 1-4.

Boyle, S.A., Lourenço, W.C., Silva, L.R. & Smith, A.T. (2009) Travel and spatial patterns change when *Chiropotes satanas chiropotes* inhabit forest fragments. *International Journal of Primatology*, **30**, 515-531.

Boyle, S.A., Smith, A.T. (2010) Can landscape and species characteristics predict primate presence in forest fragments in the Brazilian Amazon? *Biological Conservation*, **143**, 1134-1143.

Boyle, S.A., Smith, A.T. (2010) Behavioral modifications in northern bearded saki monkeys (*Chiropotes satanas chiropotes*) in forest fragments of central Amazonia. *Primates*, **51**, 43-51.

Boyle, S.A., Zartman, C.E., Spironello, W.R. & Smith, A.T. (2012) Implications of habitat fragmentation on the diet of bearded saki monkeys in central Amazonian forest. *Journal of Mammalogy*, **93(4)**, 959-976.

Cabral, M.M.M., Mattos, G.E. & Rosas, F.C.W. (2008) Mammals, birds and reptiles in Balbina reservoir, state of Amazonas, Brazil. *Check List*, **4(2)**, 152-158.

Camargo, C.C. & Ferrari, S.F. (2007) Interactions between tayras (*Eira barbara*) and red-handed howlers (*Alouatta belzebul*) in eastern Amazonia. *Primates*, **48**, 147-150.

Carvalho-Jr., O. (2003) Primates in a forest fragment in eastern Amazonia. *Neotropical Primates*, **11(2)**, 100-103.

Ferrari, S.F., Bobadilla, U.L. & Emidio-Silva, C. (2007) Where have all the titis gone? The heterogeneous distribution of *Callicebus moloch* in eastern Amazonia, and its implications for the conservation of Amazonian primates. *Primate Conservation*, **22**, 49-54.

Fialho, M.S. (2010) Contribuição à Distribuição do Gênero *Mico*, (Callitrichidae, Primates) no Médio Teles Pires, Jacareacanga, Pará. *Neotropical Primates*, **17(1)**, 31-32.

Lenz, B.B. & Reis, A. M. (2011) Harpy Eagle-Primate Interactions in the Central Amazon. *The Wilson Journal of Ornithology*, **123(2)**, 404-408.

Martins, S.S., Lima, E.M. & Silva-Júnior, J.S. (2005) Predation of a bearded saki (*Chiropotes utahicki*) by a harpy eagle (*Harpia harpyja*). *Neotropical Primates*, **13(1)**, 7-10.

Menezes-Costa, A., Machado-Ferreira, E., Voloch, C.M., Bonvicino, C.R., Seuánez, H.N., Leoncini, O. & Soares, C.A.G. (2013) Identification of Bacterial Infection in Neotropical Primates. *Microbial* *Ecology*, **66**, 471-478.

Oliveira, A.C.M. & Ferrari, S.F. (2000) Seed dispersal by black-handed tamarins, *Saguinus midas niger* (Callitrichinae, Primates): implications for the regeneration of degraded forest habitats in eastern Amazonia. *Journal of Tropical Ecology*, **16**, 709-716.

Oliveira, L.C., Loretto, D., Viana, L.R., Silva-Jr., J.S. & Fernandes, W.G. (2009) Primate community of the tropical rain forests of Saracá-Taqüera National Forest, Pará, Brazil. *Brazilian Journal of Biology*, **69(3)**, 631-637.

Oliveira, M.L., Baccaro, F.B., Braga-Neto, R. & Magnusson, W.E. (2008) *Reserva Ducke: A biodiversidade amazônica através de uma grade*. Áttema Design Editorial, Manaus.

Peres, C.A. & Nascimento, H.S. (2006) Impact of game hunting by the Kayapó of south-eastern Amazonia: implications for wildlife conservation in tropical forest indigenous reserves. *Biodiversity and Conservation*, **15**, 2627-2653.

Pimenta, F.E. & Silva-Júnior, J.S. (2005) An update on the distribution of Primates of the Tapajós-Xingu interfluvium, Central Amazonia. *Neotropical Primates*, **13(2)**, 23-28.

Pinheiro, T., Ferrari, S.F. & Lopes, M.A. (2011) Polyspecific Associations Between Squirrel Monkeys (*Saimiri sciureus*) and Other Primates in Eastern Amazonia. *American Journal of Primatology*, **73**, 1145-1151.

Pinheiro, T., Ferrari, S.F. & Lopes, M.A. (2013) Activity budget, diet, and use of space by two groups of squirrel monkeys (*Saimiri sciureus*) in eastern Amazonia. *Primates*, **54**, 301-308.

Port-Carvalho, M. & Ferrari, S.F. (2004) Occurrence and diet of the black bearded saki (*Chiropotes satanás satanas*) in the fragmented landscape of western Maranhão, Brazil. *Neotropical Primates*, **12(1)**, 17-21.

Rodrigues, L.F. & Vidal, M.D. (2011) Densidade e Tamanho Populacional de Primatas em uma Área de Terra Firme na Amazônia Central. *Neotropical Primates*, **18(1)**, 9-16.

Silva, F.H.A. (2007) *Dieta do gavião-real Harpia harpyja (Aves: Accipitridae) em florestas de terra firme de Parintins, Amazonas, Brasil*. Dissertação de mestrado, Programa de Pós-Graduação em Biologia Tropical e Recursos Naturais, Universidade Federal do Amazonas.

Silva, S.S.B. & Ferrari, S.F. (2009) Behavior patterns of southern bearded sakis (*Chiropotes satanas*) in the fragmented landscape of eastern Brazilian Amazonia. *American Journal of Primatology*, **71**, 1-7.

Silva-Júnior, J.S., Figueiredo-Ready, W.M.B. & Ferrari, S.F. (2013) Taxonomy and geographic distribution of the Pitheciidae. *Evolutionary biology and conservation of Titis, Sakis and Uacaris* (ed. by L.M. Veiga, A.A. Barnett, S.F. Ferrari and M.A. Norconk), pp. 31-42. Cambridge University Press, Cambridge.

Stone, A.I., Lima, E.M., Aguiar, G.F.S., Camargo, C.C., Flores, T.A., Kelt, D.A., Marques-Aguiar, S.A., Queiroz, J.A.L., Ramos, R.M. & Silva Júnior, J.S. (2009) Non-volant mammalian diversity in fragments in extreme eastern Amazonia. *Biodiversity and Conservation*, **18**, 1685-1694.

Vaz, S.M. (2001) Primatas da região do rio Tapajós, Pará, Brasil. *Neotropical Primates*, **9(2)**,54-57.

Veiga, L.M. (2006) *Ecologia e comportamento do cuxiú-preto (Chiropotes satanas) na paisagem fragmentada da Amazônia Oriental*. Tese de doutorado, Programa de Pós-Graduação em Teoria e Pesquisa do Comportamento, UFPA.

Veiga, L.M. & Ferrari, S.F. (2006) Predation of arthropods by southern bearded sakis (*Chiropotes satanás*) in eastern Brazilian Amazonia. *American Journal of Primatology*, **68**, 209-215.

Veiga, L.M. & Ferrari, S.F. (2007) Geophagy at termitaria by bearded sakis (*Chiropotes satanas*) in southeastern Brazilian Amazonia. *American Journal of Primatology*, **69**, 816-820.

Vulinec, K., Lambert, J.E. & Mellow, D.J. (2006) Primate and dung beetle communities in secondary growth rain forests: implications for conservation of seed dispersal systems. *International Journal of Primatology*, **27(3)**, 855-879.

***Lagothrix***

Ferrari, S.F., Cruz Neto, E.H., Iwanaga, S. & Corrêa, H.K.M. (1996) An unusual primate community at the Estação Ecológica Serra dos Três Irmãos, Rondônia, Brazil. *Neotropical Primates*, **4(2)**, 55-56.

Haugaasen, T. & Peres, C.A. (2005) Mammal assemblage structure in Amazonian flooded and unflooded forests. *Journal of Tropical Ecology*, **21**, 133-145.

Haugaasen, T. & Peres, C.A. (2007) Vertebrate responses to fruit production in Amazonian flooded and unflooded forests. *Biodiversity and Conservation,* **16**, 4165-4190.

Haugaasen, T. & Peres, C.A. (2009) Interspecific primate associations in Amazonian flooded and unflooded forests. *Primates*, **50**, 239-251.

Iwanaga, S. & Ferrari, S.F. (2002) Geographic distribution and abundance of woolly (*Lagothrix cana*) and spider (*Ateles chamek*) monkeys in southwestern brazilian Amazonia. *American Journal of Primatology*, **56**, 57-64.

Iwanaga, S. & Ferrari, S.F. (2002) Geographic distribution of red howlers (*Alouatta seniculus*) in southwestern Brazilian Amazonia, with notes on *Alouatta caraya*. *International Journal of Primatology*, **23(6)**, 1245-1256.

Kasecker, T.P. (2006) *Efeito da estrutura do habitat sobre a riqueza e composição de comunidades de primatas da RDS Piagaçu-Purus, Amazônia Central, Brasil*. Dissertação de mestrado, Programa de Pós-Graduação em Biologia Tropical e Recursos Naturais, Universidade Federal do Amazonas.

Levi, T. & Peres, C.A. (2013) Dispersal vacuum in the seedling recruitment of a primate-dispersed Amazonian tree. *Biological Conservation*, **163**, 99-106.

Lopes, M.A.O.A. & Rehg, J.A. (2003) Observations of *Callimico goeldii* with *Saguinus imperator* in the Serra do Divisor National Park, Acre, Brazil. *Neotropical Primates*, **11(3)**, 181-183.

Peres, C.A. (1988) Primate community structure in western Brazilian Amazonia. *Primate Conservation* **9**, 83-86.

Rylands, A.B., Schneider, H., Langguth, A., Mittermeier, R.A., Groves, C.P. & Rodríguez-Luna, E. (2000) An assessment of the diversity of New World primates. *Neotropical Primates*, **8(2)**, 61-93.

***Mico***

Barlow, J. & Peres, C.P. (2006) Effects of single and recurrent wildfires on fruit production and large vertebrate abundance in a central Amazonian forest. *Biodiversity and Conservation*, **15**, 985-1012.

Bobadilla, U.L. & Ferrari, S.F. (2000) Habitat use by *Chiropotes satanas utahicki* and syntopic Platyrrhines in eastern Amazonia. *American Journal of Primatology*, **50**, 215-224.

Ferrari, S.F. (1993) An update on the black-headed marmoset, *Callithrix nigriceps* Ferrari and Lopes 1992. *Neotropical Primates*, **1(4)**, 11-13.

Ferrari, S.F. & Lopes, M.A. (1992) A new species of marmoset, genus Callithrix Erxleben, 1777 (Callitrichidae, Primates), from western Brazilian Amazonia. *Goeldiana Zoologia*, **12**, 1-12.

Ferrari, S.F., Sena, L., Schneider, M.P. & Silva-Júnior, J.S. (2010) Rondon’s marmoset, *Mico rondoni* sp.n., from southwestern Brazilian Amazonia. *International Journal of Primatology*, **31**, 693-714.

Ferrari, S.F., Coutinho, P.E.G. & Corrêa, H.K.M. (2010) Congenital digital aplasia in a free-ranging group of silvery marmosets, *Mico argentatus*. *Journal of Medical Primatology*, **39**, 166-169.

Fialho, M.S. (2010) Contribuição à Distribuição do Gênero *Mico*, (Callitrichidae, Primates) no Médio Teles Pires, Jacareacanga, Pará. *Neotropical Primates*, **17(1)**, 31-32.

Garbino, G.S.T. (2011) The Southernmost Record of *Mico emiliae* (Thomas, 1920) for the State of Mato Grosso, Northern Brazil. *Neotropical Primates*, **18(2)**, 53-55.

Martins, S.S., Lima, E.M. & Silva-Júnior, J.S. (2005) Predation of a bearded saki (*Chiropotes utahicki*) by a harpy eagle (*Harpia harpyja*). *Neotropical Primates*, **13(1)**, 7-10.

Noronha, M.A., Silva-Júnior, J.S., Spironello, W.R. & Ferreira, D.C. (2008) New occurrence records of maués marmoset, *Mico mauesi* (Primates, Callitrichidae). *Neotropical Primates*, **15(1)**, 24-26.

Noronha, M.A., Spironello, W.R. & Ferreira, D.C. (2007) New occurrence records and eastern extension to the range of *Callicebus cinerascens* (Primates, Pitheciidae). *Neotropical Primates*, **14(3)**, 137-139.

Pimenta, F.E. & Silva-Júnior, J.S. (2005) An update on the distribution of Primates of the Tapajós-Xingu interfluvium, Central Amazonia. *Neotropical Primates*, **13(2)**, 23-28.

Röhe, F. & Silva-Júnior, J.S. (2009) Confirmation of *Callicebus dubius* (Pitheciidae) Distribution and Evidence of Invasion into the Geographic Range of *Callicebus stephennashi*. *Neotropical Primates*, **16(2)**, 71-73.

Rylands, A.B., Schneider, H., Langguth, A., Mittermeier, R.A., Groves, C.P. & Rodríguez-Luna, E. (2000) An assessment of the diversity of New World primates. *Neotropical Primates*, **8(2)**, 61-93.

Rylands, A.B., Mittermeier, R.A. & Silva Jr., J.S. (2012) Neotropical primates: taxonomy and recently described species and subspecies. *International Zoo Yearbook*, **46**, 11-24.

Rylands, A.B. & Mittermeier, R.A. (2009) The diversity of the New World primates (Platyrrhini). *South American primates: Comparative perspectives in the study of bahavior, ecology, and conservation* (ed. by P.A. Garber, A. Estrada, J.C. Bicca-Marques, E.W. Heymann and K.B. Strier), pp. 23-54. Springer, New York.

Silva-Júnior, J.S. & Noronha, M.A. (1998) On a new species of bare-eared marmoset, Genus *Callithrix* Erxleben, 1777, from Central Amazonia, Brazil (Primates: Callitrichidae). *Goeldiana Zoologia*, **21**, 1-28.

Tagliaro, C.H., Schneider, M.P.C., Schneider, H., Sampaio, I. & Stanhope, M. (2000) Molecular studies of *Callithrix pygmaea* (Primates, Platyrrhini) based on transferring intronic and ND1 regions: implications for taxonomy and conservation. *Genetics and Molecular Biology*, **23(4)**, 729-737.

van Roosmalen, M.G.M., van Roosmalen, T., Mittermeier, R.A. & Rylands, A.B. (2000) Two new species of marmoset, genus *Callithrix* Erxleben, 1777 (Callitrichidae, Primates), from the Tapajós/Madeira interfluvium, South Central Amazonia, Brazil. *Neotropical Primates*, **8(1)**, 2-18.

Vaz, S.M. (2001) Primatas da região do rio Tapajós, Pará, Brasil. *Neotropical Primates*, **9(2)**, 54-57.

***Pithecia***

Azevedo, R.B. (2006) *Ecologia cognitiva e forrageio social em Saguinus bicolor (Spix, 1823)*. Dissertação de mestrado, Programa de Pós-Graduação em Zoologia, Pontifícia Universidade Católica do Rio Grande do Sul.

Barlow, J. & Peres, C.P. (2006) Effects of single and recurrent wildfires on fruit production and large vertebrate abundance in a central Amazonian forest. *Biodiversity and Conservation*, **15**, 985-1012.

Barnett, A.A., Boyle, S.A., Norconk, M.M., Palminteri, S., Santos, R.R., Veiga, L.M. Alvim, T.H.G., Bowler, M., Chism, J., Di Fiore, A., Fernandez-Duque, E., Guimarães, A.C.P., Harrison-Levine, A., Haugaasen, T., Lehman, S., Mackinnon, K.C., Melo, F.R., Moreira, L.S., Moura, V.S., Phillips, C.R., Pinto, L.P., Port-Carvalho, M., Setz, E.Z.F., Shaffer, C., Silva, L.R., Silva, S.S.B., Soares, R.F., Thompson, C.L., Vieira, T.M., Vreedzaam, A., Walker-Pacheco, S.E., Spironello, W.R., Maclarnon, A. & Ferrari, S.F. Terrestrial Activity In Pitheciins (Cacajao, Chiropotes, And Pithecia). *American Journal of Primatology* **74**: 1106-1127.

Bezerra, B.M., Barnett, A.A., Souto, A. & Jones, G. (2011) Ethogram and natural history of Golden-backed Uakaris (*Cacajao melanocephalus*). *International Journal of Primatology*, **32**, 46-68.

Botelho, A.L.M., Calouro, A.M., Borges, L.H.M. & Chaves, W.A. (2012) Large and medium-sized mammals of the Humaitá Forest Reserve, southwestern Amazonia, state of Acre, Brazil. *Checklist*, **8(6)**, 1190-1195.

Boyle, S.A., Lourenço, W.C., Silva, L.R. & Smith, A.T. (2009) Travel and spatial patterns change when *Chiropotes satanas chiropotes* inhabit forest fragments. *International Journal of Primatology*, **30**, 515-531.

Boyle, S.A., Smith, A.T. (2010) Can landscape and species characteristics predict primate presence in forest fragments in the Brazilian Amazon? *Biological Conservation*, **143**, 1134-1143.

Boyle, S.A., Smith, A.T. (2010) Behavioral modifications in northern bearded saki monkeys (*Chiropotes satanas chiropotes*) in forest fragments of central Amazonia. *Primates*, **51**, 43-51.

Cabral, M.M.M., Mattos, G.E. & Rosas, F.C.W. (2008) Mammals, birds and reptiles in Balbina reservoir, state of Amazonas, Brazil. *Check List*, **4(2)**, 152-158.

Calouro, A.M. (2005) *Análise do manejo florestal de “baixo impacto” e da caça de subsistência sobre uma comunidade de primatas na Floresta Estadual do Antimary (Acre, Brasil)*. Tese de doutorado, Programa de Pós-Graduação em Ecologia e Recursos Naturais, Universidade Federal de São Carlos.

Ferrari, S.F., Cruz Neto, E.H., Iwanaga, S. & Corrêa, H.K.M. (1996) An unusual primate community at the Estação Ecológica Serra dos Três Irmãos, Rondônia, Brazil. *Neotropical Primates*, **4(2)**, 55-56.

Garber, P.A. & Leigh, S.R. (2001) Patterns of positional behavior in mixed-species troops of *Callimico goeldii*, *Saguinus labiatus*, and *Saguinus fuscicollis* in northwestern Brazil. *American Journal of Primatology*, **54**, 17-31.

Haugaasen, T. & Peres, C.A. (2005) Mammal assemblage structure in Amazonian flooded and unflooded forests. *Journal of Tropical Ecology*, **21**, 133-145.

Haugaasen, T. & Peres, C.A. (2007) Vertebrate responses to fruit production in Amazonian flooded and unflooded forests. *Biodiversity and Conservation*, **16**, 4165-4190.

Haugaasen, T. & Peres, C.A. (2009) Interspecific primate associations in Amazonian flooded and unflooded forests. *Primates*, **50**, 239-251.

Hershkovitz, P. (1987) Uacaries, New World monkeys of the genus *Cacajao* (Cebidae, Platyrrhini): a preliminary taxonomic review with the description of a new subspecies. *American Journal of Primatology*, **12**, 1-53.

Kasecker, T.P. (2006) *Efeito da estrutura do habitat sobre a riqueza e composição de comunidades de primatas da RDS Piagaçu-Purus, Amazônia Central, Brasil*. Dissertação de mestrado, Programa de Pós-Graduação em Biologia Tropical e Recursos Naturais, Universidade Federal do Amazonas.

Lopes, M.A.O.A. & Rehg, J.A. (2003) Observations of *Callimico goeldii* with *Saguinus imperator* in the Serra do Divisor National Park, Acre, Brazil. *Neotropical Primates*, **11(3)**, 181-183.

Oliveira, L.C., Loretto, D., Viana, L.R., Silva-Jr., J.S. & Fernandes, W.G. (2009) Primate community of the tropical rain forests of Saracá-Taqüera National Forest, Pará, Brazil. *Brazilian Journal of Biology*, **69(3)**, 631-637.

Oliveira, M.L., Baccaro, F.B., Braga-Neto, R. & Magnusson, W.E. (2008) *Reserva Ducke: A biodiversidade amazônica através de uma grade*. Áttema Design Editorial, Manaus.

Parry, L., Barlow, J. & Peres, C.A. (2007) Large-vertebrate assemblages of primary and secondary forests in the Brazilian Amazon. *Journal of Tropical Ecology*, **23**, 653-662.

Parry, L., Barlow, J. & Peres, C.A. (2009) Allocation of hunting effort by Amazonian smallholders: Implications for conserving wildlife in mixed-use landscapes. *Biological Conservation*, **142**, 1777-1786.

Peres, C.A. (1988) Primate community structure in western Brazilian Amazonia. *Primate Conservation*, **9**. 83-86.

Rehg, J.A. (2006) Seasonal variation in polyspecific associations among *Callimico goeldii*, *Saguinus labiatus*, and *S. fuscicollis* in Acre, Brazil. *International Journal of Primatology*, **27(5)**, 1399-1428.

Rodrigues, L.F. & Vidal, M.D. (2011) Densidade e Tamanho Populacional de Primatas em uma Área de Terra Firme na Amazônia Central. *Neotropical Primates*, **18(1)**, 9-16.

Rylands, A.B., Schneider, H., Langguth, A., Mittermeier, R.A., Groves, C.P. & Rodríguez-Luna, E. (2000) An assessment of the diversity of New World primates. *Neotropical Primates*, **8(2)**, 61-93.

Rylands, A.B., Mittermeier, R.A. & Silva Jr., J.S. (2012) Neotropical primates: taxonomy and recently described species and subspecies. *International Zoo Yearbook*, **46**, 11-24.

Rylands, A.B. & Mittermeier, R.A. (2009) The diversity of the New World primates (Platyrrhini). *South American primates: Comparative perspectives in the study of behavior, ecology, and conservation* (ed. by P.A. Garber, A., Estrada, J.C. Bicca-Marques, E.W. Heymann and K.B. Strier), pp. 23-54. Springer, New York.

Sampaio, R., Hack, R.O.E., Aguiar, K.M.O., Kuniy, A.A. & Silva-Júnior, J.A. (2012) Correção da distribuição Geográfica Do Parauacu-Cinza (*Pithecia irrorata* Gray 1842) No Limite Sudoeste da Amazônia Brasileira. *Neotropical Primates*, **19(1)**, 34-36.

Setz, E.Z.F., Enzweiler, J., Solferini, V.N., Amêndola, M.P. & Berton, R.S. (1999) Geophagy in the golden-faced saki monkey (*Pithecia pithecia chrysocephala*) in the central Amazon. *Journal of Zoology*, **247**, 91-103.

Silva, F.H.A. (2007) *Dieta do gavião-real Harpia harpyja (Aves: Accipitridae) em florestas de terra firme de Parintins, Amazonas, Brasil*. Dissertação de mestrado, Programa de Pós-Graduação em Biologia Tropical e Recursos Naturais, Universidade Federal do Amazonas.

Vulinec, K., Lambert, J.E. & Mellow, D.J. (2006) Primate and dung beetle communities in secondary growth rain forests: implications for conservation of seed dispersal systems. *International Journal of Primatology*, **27(3)**, 855-879.

***Saguinus***

Azevedo, R.B. (2006) *Ecologia cognitiva e forrageio social em Saguinus bicolor (Spix, 1823)*. Dissertação de mestrado, Programa de Pós-Graduação em Zoologia, Pontifícia Universidade Católica do Rio Grande do Sul.

Bezerra, B.M., Barnett, A.A., Souto, A. & Jones, G. (2011) Ethogram and natural history of Golden-backed Uakaris (*Cacajao melanocephalus*). *International Journal of Primatology*, **32**, 46-68.

Bicca-Marques, J.C. & Garber, P.A. (2004) Use of spatial, visual, and olfactory information during foraging in wild nocturnal and diurnal anthropoids: A field experiment comparing *Aotus*, *Callicebus*, and *Saguinus*. *American Journal of Primatology*, **62**, 171-187.

Bicca-Marques, J.C. & Garber, P.A. (2005) Use of social and ecological information in tamarin foraging decisions. *International Journal of Primatology*, **26(6)**, 1321-1344.

Bobadilla, U.L. & Ferrari, S.F. (2000) Habitat use by *Chiropotes satanas utahicki* and syntopic Platyrrhines in eastern Amazonia. *American Journal of Primatology*, **50**, 215-224.

Botelho, A.L.M., Calouro, A.M., Borges, L.H.M. & Chaves, W.A. (2012) Large and medium-sized mammals of the Humaitá Forest Reserve, southwestern Amazonia, state of Acre, Brazil. *Checklist*, **8(6)**, 1190-1195.

Boyle, S.A., Lourenço, W.C., Silva, L.R. & Smith, A.T. (2009) Travel and spatial patterns change when *Chiropotes satanas chiropotes* inhabit forest fragments. *International Journal of Primatology*, **30**, 515-531.

Boyle, S.A., Smith, A.T. (2010) Can landscape and species characteristics predict primate presence in forest fragments in the Brazilian Amazon? *Biological Conservation*, **143**, 1134-1143.

Boyle, S.A., Smith, A.T. (2010) Behavioral modifications in northern bearded saki monkeys (*Chiropotes satanas chiropotes*) in forest fragments of central Amazonia. *Primates*, **51**, 43-51.

Cabral, M.M.M., Mattos, G.E. & Rosas, F.C.W. (2008) Mammals, birds and reptiles in Balbina reservoir, state of Amazonas, Brazil. *Check List*, **4(2)**, 152-158.

Calleia, F.O., Rohe, F. & Gordo, M. (2009) Hunting Strategy of the Margay (*Leopardus wiedii*) to Attract the Wild Pied Tamarin (*Saguinus bicolor*). *Neotropical Primates*, **16(1)**, 32-34.

Calouro, A.M. (2005) *Análise do manejo florestal de “baixo impacto” e da caça de subsistência sobre uma comunidade de primatas na Floresta Estadual do Antimary (Acre, Brasil)*. Tese de doutorado, Programa de Pós-Graduação em Ecologia e Recursos Naturais, Universidade Federal de São Carlos.

Camargo, C.C. & Ferrari, S.F. (2007) Interactions between tayras (*Eira barbara*) and red-handed howlers (*Alouatta belzebul*) in eastern Amazonia. *Primates*, **48**, 147-150.

Carvalho-Jr., O. (2003) Primates in a forest fragment in eastern Amazonia. *Neotropical Primates*, **11(2)**, 100-103.

Dominy, N.J., Garber, P.A., Bicca-Marques, J.C. & Azevedo-Lopes, M.A. (2003) Do female tamarins use visual cues to detect fruit rewards more successfully than do males? *Animal Behaviour*, **66**, 829-837.

Ferrari, S.F., Cruz Neto, E.H., Iwanaga, S. & Corrêa, H.K.M. (1996) An unusual primate community at the Estação Ecológica Serra dos Três Irmãos, Rondônia, Brazil. *Neotropical Primates*, **4(2)**, 55-56.

Ferrari, S.F., Bobadilla, U.L. & Emidio-Silva, C. (2007) Where have all the titis gone? The heterogeneous distribution of *Callicebus moloch* in eastern Amazonia, and its implications for the conservation of Amazonian primates. *Primate Conservation*, **22**, 49-54.

Ferrari, S.F., Sena, L., Schneider, M.P. & Silva-Júnior, J.S. (2010) Rondon’s marmoset, *Mico rondoni* sp.n., from southwestern Brazilian Amazonia. *International Journal of Primatology*, **31**, 693-714.

Garber, P.A. & Leigh, S.R. (2001) Patterns of positional behavior in mixed-species troops of *Callimico goeldii*, *Saguinus labiatus*, and *Saguinus fuscicollis* in northwestern Brazil. *American Journal of Primatology*, **54**, 17-31.

Groves, C.P. (2005) Order Primates. *Mammal species of the world: A taxonomic and geographic reference, Vol. 1*, (ed. by D.E. Wilson and D.M. Reeder), pp. 111-184. Johns Hopkins University Press, Baltimore.

Groves, C.P. (2001) *Primate Taxonomy*. Smithsonian Institute Press, Washington, D.C.

Haugaasen, T. & Peres, C.A. (2005) Mammal assemblage structure in Amazonian flooded and unflooded forests. *Journal of Tropical Ecology*, **21**, 133-145.

Haugaasen, T. & Peres, C.A. (2007) Vertebrate responses to fruit production in Amazonian flooded and unflooded forests. *Biodiversity and Conservation*, **16**, 4165-4190.

Haugaasen, T. & Peres, C.A. (2009) Interspecific primate associations in Amazonian flooded and unflooded forests. *Primates*, **50**, 239-251.

Kasecker, T.P. (2006) *Efeito da estrutura do habitat sobre a riqueza e composição de comunidades de primatas da RDS Piagaçu-Purus, Amazônia Central, Brasil*. Dissertação de mestrado, Programa de Pós-Graduação em Biologia Tropical e Recursos Naturais, Universidade Federal do Amazonas.

Levi, T. & Peres, C.A. (2013) Dispersal vacuum in the seedling recruitment of a primate-dispersed Amazonian tree. *Biological Conservation*, **163**, 99-106.

Lopes, M.A.O.A. & Rehg, J.A. (2003) Observations of *Callimico goeldii* with *Saguinus imperator* in the Serra do Divisor National Park, Acre, Brazil. *Neotropical Primates*, **11(3)**, 181-183.

Martins, S.S., Lima, E.M. & Silva-Júnior, J.S. (2005) Predation of a bearded saki (*Chiropotes utahicki*) by a harpy eagle (*Harpia harpyja*). *Neotropical Primates*, **13(1)**, 7-10.

Mittermeier, R.A. & Wallace, R.B. (2008) *Saguinus labiatus*, in: IUCN 2013. IUCN Red List of Threatened Species. Version 2013.1. <www.iucnredlist.org>

Nunes, C.A., Bicca-Marques, J.C., Schacht, K. & Araripe, A.C.A. (1998) Reaction of wild emperor tamarins to the presence of a snak. *Neotropical Primates*, **6(1)**, 20.

Oliveira, A.C.M. & Ferrari, S.F. (2000) Seed dispersal by black-handed tamarins, *Saguinus midas niger* (Callitrichinae, Primates): implications for the regeneration of degraded forest habitats in eastern Amazonia. *Journal of Tropical Ecology*, **16**, 709-716.

Oliveira, A.C.M. & Ferrari, S.F. (2008) Habitat exploitation by free-ranging *Saguinus niger* in eastern Amazonia. *International Journal of Primatology*, **29**, 1499-1510.

Oliveira, L.C., Loretto, D., Viana, L.R., Silva-Jr., J.S. & Fernandes, W.G. (2009) Primate community of the tropical rain forests of Saracá-Taqüera National Forest, Pará, Brazil. *Brazilian Journal of Biology*, **69(3)**, 631-637.

Oliveira, L.C., Mendel, S.M., Silva-Júnior, J.S. & Fernandes, G.W. (2004) New records of Martins’ bare-face tamarin, *Saguinus martinsi* (Primates: Callitrichidae). *Neotropical Primates*, **12(1)**, 9-12.

Oliveira, M.L., Baccaro, F.B., Braga-Neto, R. & Magnusson, W.E. (2008) *Reserva Ducke: A biodiversidade amazônica através de uma grade*. Áttema Design Editorial, Manaus.

Parry, L., Barlow, J. & Peres, C.A. (2007) Large-vertebrate assemblages of primary and secondary forests in the Brazilian Amazon. *Journal of Tropical Ecology*, **23**, 653-662.

Parry, L., Barlow, J. & Peres, C.A. (2009) Allocation of hunting effort by Amazonian smallholders: Implications for conserving wildlife in mixed-use landscapes. *Biological Conservation*, **142**, 1777-1786.

Peres, C.A. (1988) Primate community structure in western Brazilian Amazonia. *Primate Conservation*, **9**, 83-86.

Pinheiro, T., Ferrari, S.F. & Lopes, M.A. (2011) Polyspecific Associations Between Squirrel Monkeys (*Saimiri sciureus*) and Other Primates in Eastern Amazonia. *American Journal of Primatology*, **73**, 1145-1151.

Pinheiro, T., Ferrari, S.F. & Lopes, M.A. (2013) Activity budget, diet, and use of space by two groups of squirrel monkeys (*Saimiri sciureus*) in eastern Amazonia. *Primates*, **54**, 301-308.

Rehg, J.A. (2006) Seasonal variation in polyspecific associations among *Callimico goeldii*, *Saguinus labiatus*, and *S. fuscicollis* in Acre, Brazil. *International Journal of Primatology*, **27(5)**, 1399-1428.

Rehg, J.A. (2010) Plant Feeding Patches: Patterns of Use by Associating *Callimico goeldii*, *Saguinus labiatus*, and *S. fuscicollis*. *Neotropical Primates*, **17(1)**, 18-21.

Rodrigues, L.F. & Vidal, M.D. (2011) Densidade e Tamanho Populacional de Primatas em uma Área de Terra Firme na Amazônia Central. *Neotropical Primates*, **18(1)**, 9-16.

Röhe, F., Silva-Júnior, J.S., Sampaio, R., Rylands, A.B. (2009) A new subspecies of *Saguinus fuscicollis* (Primates, Callitrichidae). *International Journal of Primatology*, **30**, 533-551.

Rylands, A.B., Schneider, H., Langguth, A., Mittermeier, R.A., Groves, C.P. & Rodríguez-Luna, E. (2000) An assessment of the diversity of New World primates. *Neotropical Primates*, **8(2)**, 61-93.

Rylands, A.B. & Mittermeier, R.A. (2009) The diversity of the New World primates (Platyrrhini). *South American primates: Comparative perspectives in the study of bahavior, ecology, and conservation*, (ed. by P.A. Garber, A. Estrada, J.C. Bicca-Marques, E.W. Heymann and K.B. Strier), pp. 23-54. Springer, New York.

Santos, F.G.A., Bicca-Marques, J.C., Calegaro-Marques, C., Farias, E.M.P. & Azevedo, M.A.O. (1995) On the occurrence of parasites in free-ranging callitrichids. *Neotropical Primates*, **3(2)**, 46-47.

Silva, C.R. (2007) Registro de alimentação noturna em macaco-prego (*Cebus apella*). *Neotropical Primates*, **14(2)**, 72-74.

Silva-Júnior, J.S. (1988) A range extension for *Saguinus labiatus thomasi*. *Primate Conservation*, **9**, 23-24.

Souza, L.L., Queiroz, H.L. & Ayres, J.M. (2004) The mottled-face tamarin, *Saguinus inustus*, in the Amanã Sustainable Development Reserve, Amazonas, Brazil. *Neotropical Primates*, **12(3)**, 121-122.

Stone, A.I., Lima, E.M., Aguiar, G.F.S., Camargo, C.C., Flores, T.A., Kelt, D.A., Marques-Aguiar, S.A., Queiroz, J.A.L., Ramos, R.M. & Silva Júnior, J.S. (2009) Non-volant mammalian diversity in fragments in extreme eastern Amazonia. *Biodiversity and Conservation*, **18**, 1685-1694.

Vallinoto, N., Araripe, J., Rego, P.S., Tagliaro, C.H., Sampaio, I. & Schneider, H. (2006) Tocantins river as an effective barrier to gene flow in *Saguinus niger* populations. *Genetics and Molecular Biology*, **29(2)**, 215-219.

Valsecchi, J., Vieira, T.M., Silva-Júnior, J.S., Muniz, I.C.M. & Avelar, A.A. (2010) New data on ecology and geographic distribution of *Saguinus inustus* Schwarz, 1951 (Primates, Callitrichidae). *Brazilian Journal of Biology*, **70(2)**, 229-233.

Vidal, M.D. & Cintra, R. (2006) Effects of forest structure components on the occurrence, group size and density of groups of bare-face tamarin (*Saguinus bicolor* – Primates: Callitrichinae) in Central Amazonia. *Acta Amazonica*, **36(2)**, 237-248.

Vulinec, K., Lambert, J.E. & Mellow, D.J. (2006) Primate and dung beetle communities in secondary growth rain forests: implications for conservation of seed dispersal systems. *International Journal of Primatology*, **27(3)**, 855-879.

***Saimiri***

Azevedo, R.B. (2006) *Ecologia cognitiva e forrageio social em Saguinus bicolor (Spix, 1823)*. Dissertação de mestrado, Programa de Pós-Graduação em Zoologia, Pontifícia Universidade Católica do Rio Grande do Sul.

Barlow, J. & Peres, C.P. (2006) Effects of single and recurrent wildfires on fruit production and large vertebrate abundance in a central Amazonian forest. *Biodiversity and Conservation*, **15**, 985-1012.

Bobadilla, U.L. & Ferrari, S.F. (2000) Habitat use by *Chiropotes satanas utahicki* and syntopic Platyrrhines in eastern Amazonia. *American Journal of Primatology*, **50**, 215-224.

Botelho, A.L.M., Calouro, A.M., Borges, L.H.M. & Chaves, W.A. (2012) Large and medium-sized mammals of the Humaitá Forest Reserve, southwestern Amazonia, state of Acre, Brazil. *Checklist*, **8(6)**, 1190-1195.

Cabral, M.M.M., Mattos, G.E. & Rosas, F.C.W. (2008) Mammals, birds and reptiles in Balbina reservoir, state of Amazonas, Brazil. *Check List*, **4(2)**, 152-158.

Calouro, A.M. (2005) *Análise do manejo florestal de “baixo impacto” e da caça de subsistência sobre uma comunidade de primatas na Floresta Estadual do Antimary (Acre, Brasil)*. Tese de doutorado, Programa de Pós-Graduação em Ecologia e Recursos Naturais, Universidade Federal de São Carlos.

Camargo, C.C. & Ferrari, S.F. (2007) Interactions between tayras (*Eira barbara*) and red-handed howlers (*Alouatta belzebul*) in eastern Amazonia. *Primates*, **48**, 147-150.

Carvalho-Jr., O. (2003) Primates in a forest fragment in eastern Amazonia. *Neotropical Primates*, **11(2)**, 100-103.

Dominy, N.J., Garber, P.A., Bicca-Marques, J.C. & Azevedo-Lopes, M.A. (2003) Do female tamarins use visual cues to detect fruit rewards more successfully than do males? *Animal Behaviour*, **66**, 829-837.

Ferrari, S.F., Cruz Neto, E.H., Iwanaga, S. & Corrêa, H.K.M. (1996) An unusual primate community at the Estação Ecológica Serra dos Três Irmãos, Rondônia, Brazil. *Neotropical Primates*, **4(2)**, 55-56.

Ferrari, S.F., Bobadilla, U.L. & Emidio-Silva, C. (2007) Where have all the titis gone? The heterogeneous distribution of *Callicebus moloch* in eastern Amazonia, and its implications for the conservation of Amazonian primates. *Primate Conservation*, **22**, 49-54.

Garber, P.A. & Leigh, S.R. (2001) Patterns of positional behavior in mixed-species troops of *Callimico goeldii*, *Saguinus labiatus*, and *Saguinus fuscicollis* in northwestern Brazil. *American Journal of Primatology*, **54**, 17-31.

Hershkovitz, P. (1984) Taxonomy of squirrel monkeys genus *Saimiri* (Cebidae, Platyrrhini). A preliminary report with description of a hitherto unnamed form. *American Journal of Primatology*, **7**, 155-210.

Hershkovitz, P. (1987) The taxonomy of South American sakis, genus *Pithecia* (Cebidae, Platyrrhini): a preliminary report and critical review with the description of a new species and a new subspecies. *American Journal of Primatology*, **12(4)**, 387-468.

Kasecker, T.P. (2006) *Efeito da estrutura do habitat sobre a riqueza e composição de comunidades de primatas da RDS Piagaçu-Purus, Amazônia Central, Brasil*. Dissertação de mestrado, Programa de Pós-Graduação em Biologia Tropical e Recursos Naturais, Universidade Federal do Amazonas.

Lima, E.M. & Ferrari, S.F. (2003) Diet of a free-ranging group of squirrel monkeys (*Samiri sciureus*) in eastern brazilian Amazonia. *Folia Primatologica*, **74**, 150-158.

Lopes, M.A.O.A. & Rehg, J.A. (2003) Observations of *Callimico goeldii* with *Saguinus imperator* in the Serra do Divisor National Park, Acre, Brazil. *Neotropical Primates*, **11(3)**, 181-183.

Oliveira, A.C.M. & Ferrari, S.F. (2000) Seed dispersal by black-handed tamarins, *Saguinus midas niger* (Callitrichinae, Primates): implications for the regeneration of degraded forest habitats in eastern Amazonia. *Journal of Tropical Ecology*, **16**, 709-716.

Oliveira, L.C., Loretto, D., Viana, L.R., Silva-Jr., J.S. & Fernandes, W.G. (2009) Primate community of the tropical rain forests of Saracá-Taqüera National Forest, Pará, Brazil. *Brazilian Journal of Biology*, **69(3)**, 631-637.

Paim, F.P. & Queiroz, H.L. (2009) Diferenças nos parâmetros acústicos das vocalizações de alarme das espécies de *Saimiri* Voigt, 1831 (Primates, Cebidae) na floresta de várzea – Reserva Mamirauá. *Uakari*, **5(1)**, 49-60.

Paim, F.P., Silva-Júnior, J.S., Valsecchi, J., Harada, M.L. & Queiroz, H.L. (2013) Diversity, Geographic Distribution and Conservation of Squirrel Monkeys, Saimiri (Primates, Cebidae), in the Floodplain Forests of Central Amazon. *International Journal of Primatology*, **34**, 1055-1076.

Parry, L., Barlow, J. & Peres, C.A. (2009) Allocation of hunting effort by Amazonian smallholders: Implications for conserving wildlife in mixed-use landscapes. *Biological Conservation*, **142**, 1777-1786.

Peres, C.A. (1988) Primate community structure in western Brazilian Amazonia. *Primate Conservation*, **9**, 83-86.

Pimenta, F.E. & Silva-Júnior, J.S. (2005) An update on the distribution of Primates of the Tapajós-Xingu interfluvium, Central Amazonia. *Neotropical Primates*, **13(2)**, 23-28.

Pinheiro, T., Ferrari, S.F. & Lopes, M.A. (2011) Polyspecific Associations Between Squirrel Monkeys (*Saimiri sciureus*) and Other Primates in Eastern Amazonia. *American Journal of Primatology*, **73**, 1145-1151.

Pinheiro, T., Ferrari, S.F. & Lopes, M.A. (2013) Activity budget, diet, and use of space by two groups of squirrel monkeys (*Saimiri sciureus*) in eastern Amazonia. *Primates*, **54**, 301-308.

Pontes, A.R.M. (1999) Environmental determinants of primate abundance in Maracá Island, Roraima, Brazilian Amazonia. *Journal of Zoology*, **247**, 189-199.

Rehg, J.A. (2006) Seasonal variation in polyspecific associations among *Callimico goeldii*, *Saguinus labiatus*, and *S. fuscicollis* in Acre, Brazil. *International Journal of Primatology*, **27(5)**, 1399-1428.

Rylands, A.B., Schneider, H., Langguth, A., Mittermeier, R.A., Groves, C.P. & Rodríguez-Luna, E. (2000) An assessment of the diversity of New World primates. *Neotropical Primates*, **8(2)**, 61-93.

Rylands, A.B. & Mittermeier, R.A. (2009) The diversity of the New World primates (Platyrrhini). *South American primates: Comparative perspectives in the study of bahavior, ecology, and conservation* (ed. by P.A. Garber, A. Estrada, J.C. Bicca-Marques, E.W. Heymann and K.B. Strier), pp. 23–54. Springer, New York.

Silva, C.R. (2007) Registro de alimentação noturna em macaco-prego (*Cebus apella*). *Neotropical Primates*, **14(2)**, 72-74.

Silva, S.S.B. & Ferrari, S.F. (2009) Behavior patterns of southern bearded sakis (*Chiropotes satanas*) in the fragmented landscape of eastern Brazilian Amazonia. *American Journal of Primatology*, **71**, 1-7.

Souza, L.L., Queiroz, H.L. & Ayres, J.M. (2004) The mottled-face tamarin, *Saguinus inustus*, in the Amanã Sustainable Development Reserve, Amazonas, Brazil. *Neotropical Primates*, **12(3)**, 121-122.

Stone, A.I., Lima, E.M., Aguiar, G.F.S., Camargo, C.C., Flores, T.A., Kelt, D.A., Marques-Aguiar, S.A., Queiroz, J.A.L., Ramos, R.M. & Silva Júnior, J.S. (2009) Non-volant mammalian diversity in fragments in extreme eastern Amazonia. *Biodiversity and Conservation*, **18**, 1685-1694.

Vaz, S.M. (2001) Primatas da região do rio Tapajós, Pará, Brasil. *Neotropical Primates,* **9(2)**, 54-57.

Wallace, R.B., Painter, R.L.E., Taber, A.B. & Ayres, J.M. (1996) Notes on a distributional river boundary and southern range extension for two species of Amazonian primates. *Neotropical Primates*, **4(4)**, 149-151.
