# Supplementary material for: Multi-Scales Analysis of Primate Diversity and Protected Areas at a Megadiverse Region
Source: PLoS One. 2014 Aug 18;9(8):e105205. doi: 10.1371/journal.pone.0105205 (PMC4136851; doi:10.1371/journal.pone.0105205)
Supplement: Text S2 — Specific taxonomic details followed for each genus. (DOCX) [file pone.0105205.s002.docx]

**Appendix S2:**

Specific taxonomic details followed for each genus. References cited below are listed at Text S1.

*Alouatta* – we followed the taxonomic classification according to Gregorin (2006). We also included the species *Alouatta seniculus* (Avelar, 2007). The final occurrence database for *Alouatta* includes *Allouata belzebul*, *A*. *caraya*, *A*. *discolor*, *A*. *juara*, *A*. *macconnelli*, *A*. *nigerrima*, *A*. *puruensis* and *A*. *seniculus*. The distribution database [IUCN] also includes *A. sara* and *A. ululata*.

*Aotus* - we followed the taxonomic classification according to Hershkovitz (1983). However, the species recognized in this article as *Aotus infulatus* is considered by Rylands et al. (2000, 2012) and Groves (2005) as a subspecies of *Aotus azarae* (*A. a. infulatus*). In this case we followed this last classification. The final database for *Aotus* includes *Aotus azarae*, *A*. *nancymae*, *A*. *nigriceps*, *A*. *trivirgatus* and *A*. *vociferans*.

*Ateles* – we followed the taxonomic classification according to Rylands et al. (2000, 2012), Groves (2005) and Rylands & Mittermeier (2009) that raised all Amazonian taxa to species level. These taxa have been recognized in the systematic review by Kellogg & Goldman (1944) as subespecies. The final database for *Ateles* includes *Ateles belzebuth*, *A*. *chamek*, *A*. *marginatus*, and *A*. *paniscus*.

*Cacajao* – we followed the taxonomic classification according to Boubli et al. (2008). The final database for *Cacajao* includes *Cacajao ayresi*, *C*. *calvus*, *C*. *hosomi*, and *C*. *melanocephalus*.

*Callibella* – we followed the taxonomic classification according to van Roosmalen & van Roosmalen (2003). This is a monotypic genus and the final database includes just the species *Callibella humilis*.

*Callicebus* – we followed the taxonomic classification according to van Roosmalen et al. (2002), Groves (2005), Silva-Junior et al. (2013) and Gualda-Barros et al. (2012). The final database for *Callicebus* includes *Callicebus baptista*, *C*. *bernhardi*, *C*. *brunneus*, *C*. *caligatus*, *C*. *cinerascens*, *C*. *cupreus*, *C. dubius*, C. *hoffmannsi*, *C*. *lugens*, *C*. *moloch*, *C*. *purinus*, *C*. *regulus*, *C*. *stephennashi*, *C*. *torquatus* and *C. vieirai*. The distribution database [IUCN] also includes *C. donacophilus*, *C. lucifer* and *C. pallescens*. It does not include *C. vieirai*.

*Callimico* – we followed the taxonomic classification according to Hershkovitz (1977). This is a monotypic genus and the final database includes just the species *Callimico goeldii*.

*Cebuella* - we followed the taxonomic classification according to Hershkovitz (1977). This is a monotypic genus and the final database includes just the species *Cebuella pygmaea*.

*Cebus* and *Sapajus* – we followed the taxonomic classification according to Silva-Junior (2001), updated using Alfaro et al. (2012). The final database for *Cebus* and *Sapajus* includes *Cebus albifrons*, *C*. *kaapori*, and *C*. *olivaceus*, *Sapajus* *apella*, *S*. *cay*, *S*. *libidinosus* and *S*. *macrocephalus*.

*Chiropotes* – we followed the taxonomic classification according to Silva-Junior et al. (2013). The final database for *Chiropotes* includes *Chiropotes albinasus*, *C*. *chiropotes*, *C*. *sagulatus*, *C*. *satanas*, and *C*. *utahickae*. The distribution database [IUCN] also does not include *C. sagulatus*.

*Lagothrix* – we followed the taxonomic classification according to Rylands et al. (2000). The possibility of occurrence of *L. lagotricha* in Brazil is matter for discussion. Hence the final database for *Lagothrix* includes only *Lagothrix cana*, and *L*. *poeppigii*. The distribution database [IUCN] also includes *L*. *lagotricha.*

*Mico* – we followed the taxonomic classification according to Rylands et al. (2000, 2012), van Roosmalen et al. (2000) and Rylands & Mittermeier (2009). The recently descripted species *Mico rondoni* (Ferrari et al. 2010, Rylands et al. 2012) was also included. The final database for *Mico* includes *Mico acariensis*, *M*. *argentatus*, *M*. *chrysoleucus*, *M*. *emiliae*, *M*. *humeralifer*, *M*. *intermedius*, *M*. *leucippe*, *M*. *manicorensis*, *M*. *marcai*, *M*. *mauesi*, *M*. *melanurus*, *M*. *nigriceps*, *M*. *rondoni*, and *M*. *saterei*.

*Pithecia* – we followed the taxonomic classification according to Hershkovitz (1987), Rylands et al. (2000, 2012) and Rylands & Mittermeier (2009). The final database for *Pithecia* includes *Pithecia albicans*, *P*. *irrorata*, *P*. *monachus*, and *P*. *pithecia*.

*Saguinus* – we followed the taxonomic classification according to Rylands et al. (2000). We also included the species *Saguinus melanoleucus* (Groves 2001, 2005, Rylands & Mittermeier 2009). *Saguinus labiatus thomasi* is still considered a subspecies. A new study been developed using morphologic and molecular data will probably elevate this subspecies to species level. Beyond that actually *Saguinus labiatus labiatus* and *Saguinus labiatus thomasi* have disjunct distributions (Mittermeier & Wallace 2008). For that reason, we included this subspecies in our database. The final database for *Saguinus* includes *Saguinus bicolor*, *S*. *fuscicollis*, *S*. *imperator*, *S*. *inustus*, *S*. *labiatus*, *S*. *martinsi*, *S*. *melanoleucus*, *S*. *midas*, *S*. *mystax*, *S*. *niger*, *S*. *nigricollis*, and *S*. *labiatus thomasi*. The distribution database [IUCN] also does not include *S. thomasi*.

*Saimiri* – we followed the taxonomic classification according to Rylands et al. (2000) and Rylands & Mittermeier (2009) that was based in Hershkovitz (1984, 1987b), except for *Saimiri vanzolinii*. The final database for *Saimiri* includes *Saimiri boliviensis*, *S*. *sciureus*, *S*. *ustus*, and *S*. *vanzolinii*.
